# Runx1 is a central regulator of osteogenesis for bone homeostasis by orchestrating BMP and WNT signaling pathways

**DOI:** 10.1371/journal.pgen.1009233

**Published:** 2021-01-21

**Authors:** Chen-Yi Tang, Mengrui Wu, Dongfeng Zhao, Diep Edwards, Abigail McVicar, Yuan Luo, Guochun Zhu, Yongjun Wang, Hou-De Zhou, Wei Chen, Yi-Ping Li

**Affiliations:** 1 Department of Pathology, University of Alabama at Birmingham School of Medicine, Birmingham, Alabama, United States of America; 2 Department of Metabolism & Endocrinology, Hunan provincial Key Laboratory of Metabolic Bone Diseases, National Clinical Research Center for Metabolic Diseases, The Second Xiangya Hospital, Central South University, Changsha, Hunan, China; 3 Institute of Genetics, Life Science College, Zhejiang University, Hangzhou, Zhejiang, People's Republic of China; 4 Shanghai University of Traditional Chinese Medicine, Pudong, Shanghai, China P.R; Children's Hospital of Philadelphia, UNITED STATES

## Abstract

Runx1 is highly expressed in osteoblasts, however, its function in osteogenesis is unclear. We generated mesenchymal progenitor-specific (*Runx1*^*f/f*^*Twist2-Cre)* and osteoblast-specific (*Runx1*^*f/f*^*Col1α1-Cre*) conditional knockout (Runx1 CKO) mice. The mutant CKO mice with normal skeletal development displayed a severe osteoporosis phenotype at postnatal and adult stages. Runx1 CKO resulted in decreased osteogenesis and increased adipogenesis. RNA-sequencing analysis, Western blot, and qPCR validation of Runx1 CKO samples showed that Runx1 regulates BMP signaling pathway and Wnt/β-catenin signaling pathway. ChIP assay revealed direct binding of *Runx1* to the promoter regions of *Bmp7*, *Alk3*, and *Atf4*, and promoter mapping demonstrated that *Runx1* upregulates their promoter activity through the binding regions. *Bmp7* overexpression rescued Alk3, Runx2, and Atf4 expression in *Runx1*-deficient BMSCs. Runx2 expression was decreased while Runx1 was not changed in *Alk3* deficient osteoblasts. *Atf4* overexpression in *Runx1*-deficient BMSCs did not rescue expression of Runx1, Bmp7, and Alk3. Smad1/5/8 activity was vitally reduced in Runx1 CKO cells, indicating *Runx1* positively regulates the Bmp7/Alk3/Smad1/5/8/Runx2/ATF4 signaling pathway. Notably, Runx1 overexpression in *Runx2*^*-/-*^ osteoblasts rescued expression of Atf4, OCN, and ALP to compensate *Runx2* function. *Runx1* CKO mice at various osteoblast differentiation stages reduced Wnt signaling and caused high expression of C/ebpα and Pparγ and largely increased adipogenesis. Co-culture of *Runx1*-deficient and wild-type cells demonstrated that *Runx1* regulates osteoblast−adipocyte lineage commitment both cell-autonomously and non-autonomously. Notably, *Runx1* overexpression rescued bone loss in OVX-induced osteoporosis. This study focused on the role of Runx1 in different cell populations with regards to BMP and Wnt signaling pathways and in the interacting network underlying bone homeostasis as well as adipogenesis, and has provided new insight and advancement of knowledge in skeletal development. Collectively, *Runx1* maintains adult bone homeostasis from bone loss though up-regulating Bmp7/Alk3/Smad1/5/8/Runx2/ATF4 and WNT/β-Catenin signaling pathways, and targeting *Runx1* potentially leads to novel therapeutics for osteoporosis.

## Author summary

Bone loss in osteoporosis and many other degenerative bone diseases is characterized by decreased bone formation and increased fat accumulation in the bone marrow, especially during aging, and many are suspected to have a genetic basis. Bone marrow mesenchymal stem cells (BMSCs) can differentiate into adipocytes, chondrocytes, and osteoblasts. BMSCs from aging or genetic defect subjects are less likely to differentiate into osteoblasts while more inclined to differentiate into adipocytes. However, the molecular mechanisms remain elusive. Here, we describe a new mutant mice model-*Runx1* mesenchymal progenitor-specific and osteoblast-specific conditional knockout (*Runx1* CKO) mice, which showed the severe bone formation defects and adipocytes accumulation. Here, we revealed that *Runx1* enhances osteoblast lineage commitment promotes bone formation and inhibits adipogenesis by up-regulating the Bmp7/Alk3/Smad1/5/8/Runx2/ATF4 and WNT/β-catenin signaling pathways and orchestrating multiple signaling pathways involved in bone formation. These findings further elucidated the roles of Runx1 in bone homeostasis with implications into development of novel therapeutic strategies for osteoporosis as well as other degenerative bone diseases.

## Introduction

Bone loss in osteoporosis and many other degenerative bone diseases, especially during aging, is coupled with increased adipocytes and decreased osteoblasts in the bone marrow [1–5]. These changes in cell population with age and disease are thought to occur as mesenchymal stem cells (MSCs) become more inclined to differentiate into adipocytes rather than osteoblasts [6–8]. Previous reports have suggested that the switch in lineage commitment of MSCs into adipocytes rather than osteoblasts in the bone marrow is linked to pathologic or aging conditions [6]. Differentiation of MSCs into adipocytes or osteoblasts is orchestrated by many signaling pathways and driven by different transcriptional factors [4]. Previous reports have shown that CCAAT/enhancer binding protein α (C/EBPα) and peroxisome proliferator-activated receptor γ (PPARγ or PPARG) can promote adipocyte differentiation [4, 9, 10], while *Runx2* and *Dlx5* promote osteoblast differentiation [11]. Runt-related transcription factor 1 (*Runx1*) is a DNA-binding partner of Core binding factor β (*Cbfβ*), which forms a *Runx1/Cbfβ* heterodimeric complex [12–14]. We have recently reported that *Cbfβ* is involved in osteoblast and chondrocyte differentiation as well as pathologically induced fracture healing [15–18]. However, the mechanism underlying how *Runx1* maintains osteoblast−adipocyte lineage commitment is unclear. Therefore, we deleted the *Runx1* gene at each stage of the osteoblast lineage (MSCs and early osteoblastic cells) using the *Twist2-Cre*, *Col1α1-Cre* (Mature osteoblastic cells), and *Col2α1-Cre* (Chondrocyte and osteoblastic cell) mouse lines, respectively. Our *Runx1* conditional deletion mouse models revealed that *Runx1* is critical for osteoblast lineage commitment and maintenance and *Runx1* deficiency can be an important genetic cause leading to osteoporosis.

Runx1 along with other the Runx family proteins such as *Runx2* and *Runx3* play critical role in cell fate determination [19]. As such, previous studies have demonstrated Runx1’s strong potential for the emergence of hematopoietic stem cells [20], while also involved in fracture healing [21–23]. Specifically, Runx1 functions to induce mesenchymal stem cells into early stages of chondrogenesis [22, 24]. Despite the insights gained on the roles of *Runx2* in osteoblast differentiation and skeletal development [4], the transcriptional factors that positively regulate osteoblast lineage commitment in adult, aging, or pathologic bone remain unclear. Expression of Runx1 has been detected in osteoblast progenitors, pre-osteoblasts, and mature osteoblasts. Nevertheless, the function of *Runx1* in bone homeostasis in adult stage has not been determined. *Runx2’s* interactions with multiple co-regulators and transcription factors are critical in regulation of osteoblastic lineage [25–28]. However, overexpression of *Runx2* causes negative regulation of osteoblast maturation which eventually leads to osteopenia [29]. Therefore, it is of clinical significance to determine which transcription factor(s) are able to positively regulate osteoblast cell lineage and bone homeostasis. *Runx1*, regulator of *Runx2*, is expressed in early and late stage skeletal cells, while Runx2 was initially reduced in adult skeletons, indicating *Runx1*’s importance in cell lineage determination [30]. However, the function of *Runx1* in bone formation to maintain bone homeostasis is still largely unclear.

To elucidate the roles of Runx1 in bone formation, we utilized the *Twist2 (Dermo1)*-Cre and *Col1α1-Cre* to generate the mesenchymal-specific *Runx1* conditional knockout (CKO) mice and osteoblast-specific *Runx1* CKO mice, respectively. *Runx1* deletion in the osteoblast lineage leads to a severe osteoporotic phenotype. RNA-seq analysis showed that BMP signaling was downregulated by *Runx1* deficiency in osteoblasts. We have revealed that *Runx1* is critical during postnatal bone homeostasis and skeletal development via directly binding to the *Bmp7*, *Alk3*, and *Atf4* promoters, thereby directly regulating *their* expressions. We further revealed that *Runx1* enhances osteoblast lineage commitment promotes bone formation and inhibits adipogenesis by up-regulating the Bmp7/Alk3/Smad1/5/8/Runx2/ATF4 and WNT/β-catenin signaling pathways and orchestrating multiple signaling pathways involved in bone formation. These findings further elucidated the roles of Runx1 in bone homeostasis with implications into development of novel therapeutic strategies for osteoporosis as well as other degenerative bone diseases.

## Results

### *Runx1*^*f/f*^*Twist2-Cre* and *Runx1*^*f/f*^*Col1α1-Cre* conditional knockout mice displayed a severe osteoporosis phenotype at postnatal and adult stages, but exhibited normal skeletal development

**Fig 1:**
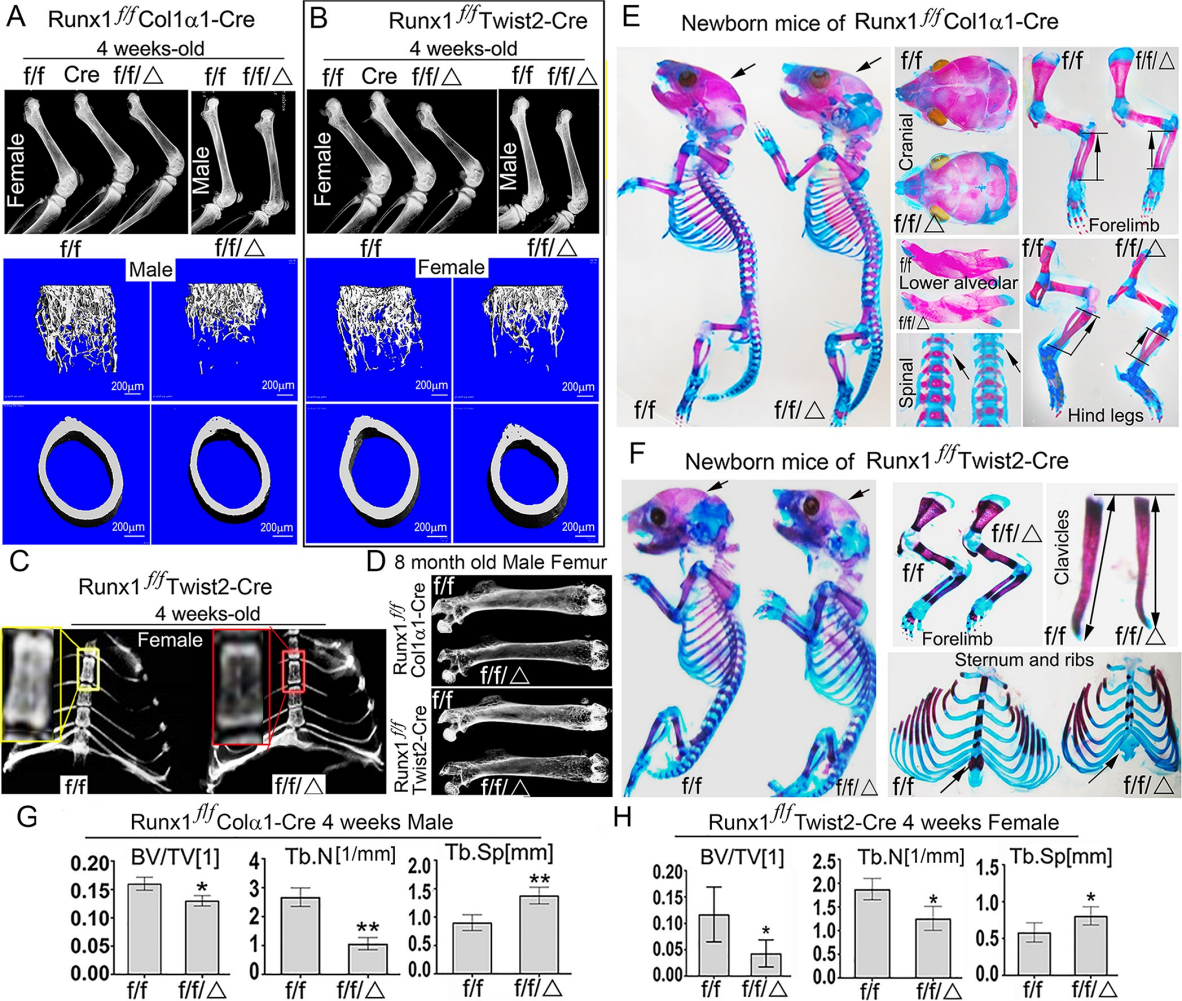
*Runx1*^*f/f*^*Twist2-Cre* and *Runx1*^*f/f*^*Col1α1-Cre* mice displayed a severe osteoporosis phenotype at postnatal and adult stages. (A, B) Radiographic images and μCT scans of (A) 4-week-old *Runx1*^*f/f*^*Col1α1-Cre* (f/f/Δ) and control (f/f) mouse tibias, and (B) 4-week-old *Runx1*^*f/f*^*Twist2-Cre* (f/f/Δ) and control (f/f) mouse femurs. (C) Radiographic images of 4-week-old *Runx1*^*f/f*^*Twist2-Cre* (f/f/Δ) and control (f/f) mouse ribs and vertebrae. (D) Radiographic images of 8-month-old *Runx1*^*f/f*^*Twist2-Cre* (f/f/Δ) and control (f/f) male mice femurs, and 8-month-old *Runx1*^*f/f*^ *Col1α1-Cre* (f/f/Δ) and control (f/f) mouse femurs. (E) Whole-mount Alizarin red and Alcian blue staining of newborn *Runx1*^*f/f*^*Col1α1-Cre* skeletons. Alizarin red and Alcian blue staining of newborn *Runx1*^*f/f*^*Col1α1-Cre* skulls, forelimbs, mandibles, hind legs, and spines. (F) Whole-mount Alizarin red and Alcian blue staining of newborn *Runx1*^*f/f*^*Twist2-Cre* skeletons. Alizarin red and Alcian blue staining of newborn *Runx1*^*f/f*^*Col1α1-Cre* forelimbs, clavicles, and sternum and ribs. (G) Quantification of bone volume/tissue volume (BV/TV), trabecular number (Tb.N), and trabecular space (Tb.Sp) of μCT scans in A. (H) Quantification of BV/TV, Tb.N, and Tb.Sp of μCT scans in B. Results are expressed as mean ± SD, n = 4 in each group. N.S, not significant. *p < 0.05, **p < 0.01, ***p < 0.001.

To investigate the role of *Runx1* in osteoblast−adipocyte lineage commitment *in vivo*, we sought to delete *Runx1* in skeletal cells at various osteoblast differentiation stages by generating *Runx1*^*f/f*^*Twist2-Cre*, *Runx1*^*f/f*^*Col1α1-Cre* and *Runx1*^*f/f*^*Col2α1-Cre* conditional knockout mice. Interestingly, radiographic analysis showed that 4-week-old male and female, 6-week-old male and female *Runx1*^*f/f*^*Col1α1-Cre* mice (Fig 1A and S1A Fig), as well as 4-week-old male and female, 6-week-old and 17-week-old male *Runx1*^*f/f*^*Twist2-Cre* mice (Fig 1B and S1B Fig) had reduced bone density compared with their control (*Runx1*^*f/f*^) littermates (Fig 1A and 1B; S1A and S1B Fig). Microcomputed tomography (μCT) analysis of the distal femora of 4-week-old *Runx1*^*f/f*^*Col1α1-Cre* and *Runx1*^*f/f*^*Twist2-Cre* mouse femurs further confirmed the reduced bone volume and trabecular bone number, as well as an increase in trabecular bone separation in the *Runx1*^*f/f*^*Col1α1-Cre* mice (Fig 1A and 1B). 4-week-old *Runx1*^*f/f*^*Col1α1-Cre* mice displayed a 40.3% reduction in bone volume/tissue volume (BV/TV), a 60.7% reduction in trabecular number (Tb.N), and a 40.2% increase in trabecular space (Tb.Sp) (Fig 1G). The 4-week-old *Runx1*^*f/f*^*Twist2-Cre* mice displayed a 50.5% reduction in BV/TV, a 30.7% reduction in Tb.N, and a 36.2% increase in Tb.Sp (Fig 1H). In addition, x-ray showed that the thoracic vertebra bone mass was noticeably decreased in 4-week-old *Runx1*^*f/f*^*Twist2-Cre* mice compared to its control (Fig 1C). X-ray of femurs from 8-month-old *Runx1*^*f/f*^*Col1α1-Cre* and *Runx1*^*f/f*^*Twist2-Cre* mice revealed a severe osteoporosis phenotype, with more bone loss in *Runx1f/fCol1α1-Cre* and *Runx1f/fTwist2-Cre* mice compared to their same litter controls (Fig 1D). Skeletons of newborn *Runx1*^*f/f*^*Col1α1-Cre* mice were severely underdeveloped as mutant skulls, calvaria, and mandibles were undecalcified with larger fontanelles (Fig 1E). Furthermore, the forelimbs and vertebrae were severely affected in the mutant mice (Fig 1E). The data suggest that bone ossification was delayed in mutant mice. Similarly, newborn *Runx1*^*f/f*^*Twist2-Cre* mice were also severely underdeveloped (Fig 1F), with undecalcified skull, larger fontanelles, undecalcified clavicles, and undecalcified sternum (Fig 1F). Using double calcein labelling to assess the mineral apposition rate, the results demonstrated that the mineral apposition rate decreased by 60.4% in the *Runx1*^*f/f*^*Col1α1-Cre* and 40.5% in the *Runx1*^*f/f*^*Twist2-Cre* mice compared with the control (S1C and S1D Fig), leading to decreased bone formation and lower bone density in the mutant mice. These data suggest that Runx1 is essential for bone ossification.

### *Runx1* deficiency impairs bone formation and increases marrow adipocyte accumulation in *Runx1*^*f/f*^*Col1α1-Cre* and *Runx1*^*f/f*^*Twist2-Cre* mice, with significantly reduced expression of osteoblast genes

**Fig 2:**
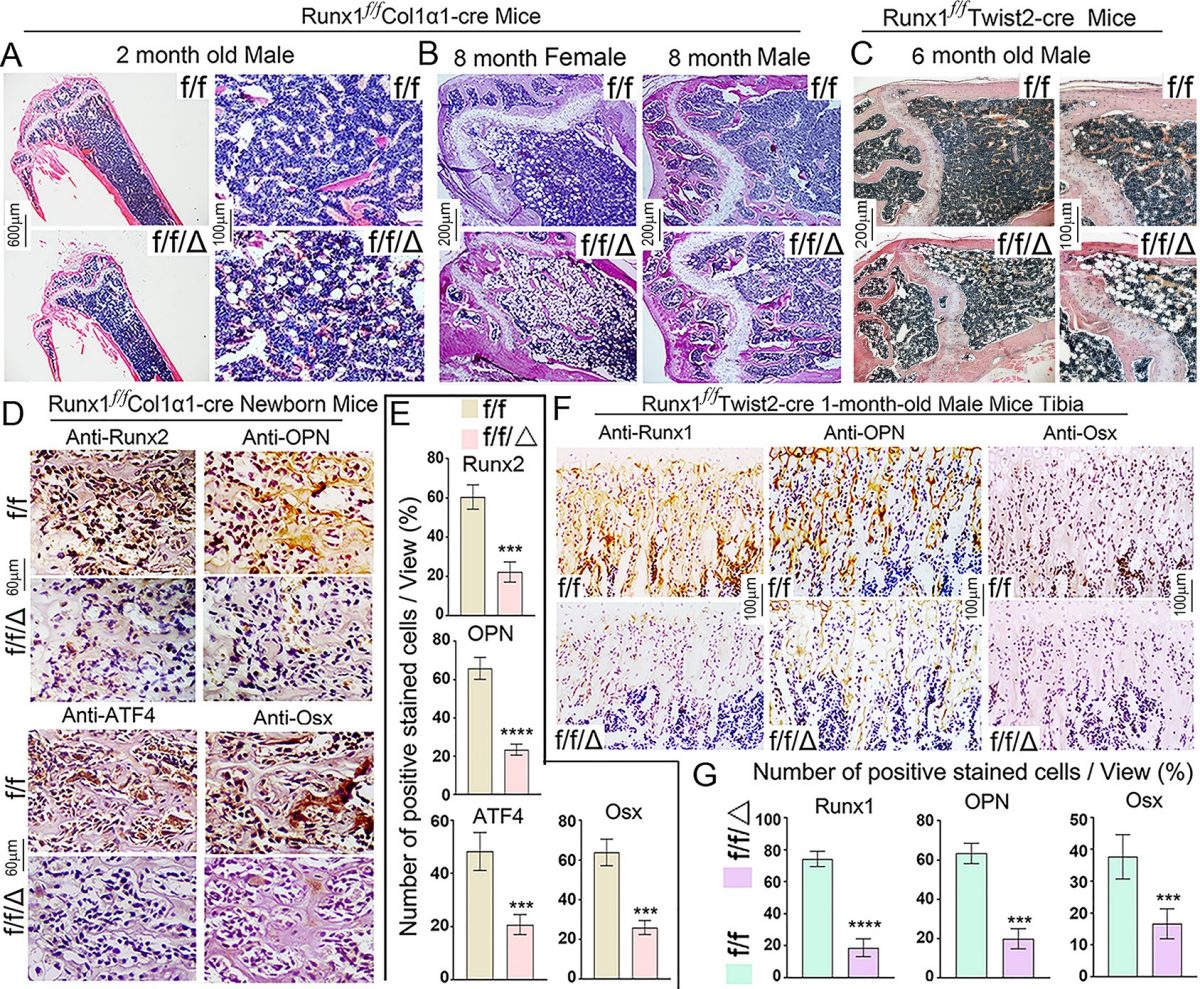
Bone formation was impaired in *Runx1*^*f/f*^*Col1α1-Cre* and *Runx1*^*f/f*^*Twist2-Cre* mice, with significantly reduced expression of osteoblast genes. (A, B) H&E stain of (A) 2-month-old and (B) 8-month-old *Runx1*^*f/f*^*Col1α1-Cre* mice femurs. (C) H&E stain of 6-month-old *Runx1*^*f/f*^*Twist2-Cre* mice femurs. (D) Immunostaining using antibodies against Runx2, Opn, Atf4, and Osx in newborn *Runx1f/fCol1α1-Cre* (f/f/Δ) and *Runx1f/f* (f/f) mouse femurs. (E) quantification of immunostaining-positive cells in D. (F) Immunostaining using antibodies against Runx1, Opn, and Osx in newborn *Runx1f/fTwist2-Cre* (f/f/Δ) and *Runx1f/f* (f/f) mouse femurs. (G) quantification of immunostaining-positive cells in F. Results are expressed as mean ± SD, n = 4 in each group. N.S, not significant; **p <0.01.

Consistent with the μ-CT results, H&E staining of 2- and 8-month-old *Runx1*^*f/f*^*Col1α1-Cre* mice displayed increased adipocytes and significantly decreased cortical bone thickness (Fig 2A and 2B), similarly, 6-month-old *Runx1*^*f/f*^*Twist2-Cre* mice also showed a significant increase in marrow adipocytes compared to control, accompanied by a decrease in the thickness of the cortical bone (Fig 2C). Femoral sections from *Runx1*^*f/f*^*Col2α1-Cre* mice were subsequently subjected to further histological analysis (S2A–S2C Fig). ALP staining showed that the osteogenesis activity in hypertrophic zone was decreased in the 3-month-old *Runx1*^*f/f*^*Col2α1-Cre* female mice femurs (S2A Fig) and the 6-month-old *Runx1*^*f/f*^*Col2α1-Cre* male mice tibias (S2C Fig) compared to their controls, which may demonstrate that chondrocyte to osteoblast commitment is compromised by *Runx1* deficiency. P1NP ELISA results showed that the osteoblast activity was compromised in *Runx1*^*f/f*^*Col1α1-Cre* and *Runx1*^*f/f*^*Twist2-Cre* mice serum (S1E Fig), but CTX-1 expression which refers to bone resorption activity was not significantly altered between the *Runx1* CKO and control mice (S1F Fig). H&E staining showed that trabecular bone number was reduced in the 3-month-old *Runx1*^*f/f*^*Col2α1-Cre* male mice femur (S2B Fig). Furthermore, we also found that adipocytes were dramatically increased in the mutant mice femur and tibia compared to their controls (S2A–S2C Fig, red arrow). We further examined the impact of *Runx1* deletion in osteoblasts through Runx2, Opn, Atf4, and Osx staining in newborn *Runx1*^*f/f*^*Col1α1-Cre* mice (Fig 2D). Immunohistochemistry staining revealed that the expression of *Runx2*, a gene important for osteoblast differentiation, was significantly decreased in *Runx1*^*f/f*^*Col1α1-Cre* mice (Fig 2A and 2E). Consistently, we found a significant decrease in the expression of Atf4, which is a gene important for osteoblast differentiation [31], in *Runx1*^*f/f*^*Col1α1-Cre* mice (Fig 2D and 2E). Further, Opn (Osteopontin) and Osx (Osterix), which are osteoblast-related genes, showed reduced expression in newborn *Runx1*^*f/f*^*Col1α1-Cre* mice trabecular bone compared with WT (Fig 2D and 2E), indicating that osteoblastogenesis may be affected in *Runx1*-deficient condition. *Runx1* deletion in MSCs through *Twist2-cre* showed similar reductions in the expression levels of Runx1, Osx, Opn, and Ocn compared to WT mice (Fig 2F and 2G). Overall, our data suggest that bone formation was decreased and adipocytes accumulation was increased in *Runx1* deficient mice.

### *Runx1* enhances osteoblast differentiation by increasing expression of regulator genes and inhibits adipogenesis via both cell-autonomous and cell-non autonomous pathways

**Fig 3:**
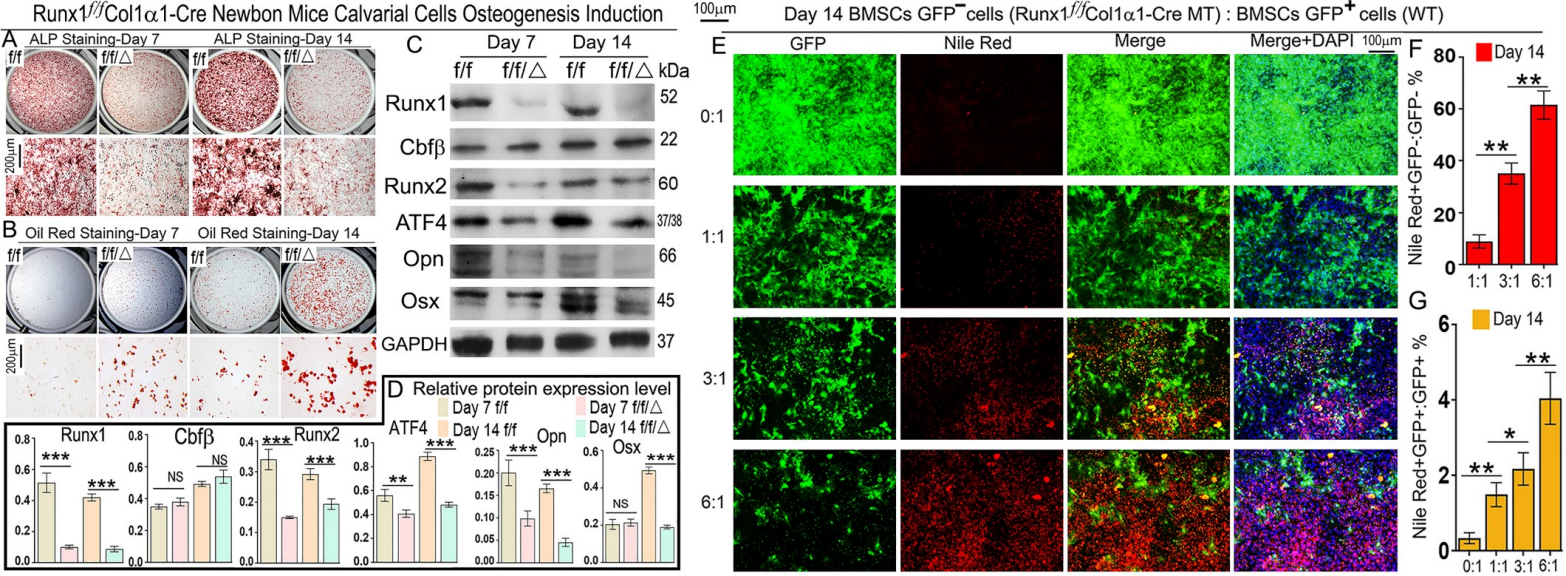
*Runx1* deficiency in primary calvarial cells cultured from *Runx1*^*f/f*^*Col1α1-Cre* mice inhibits osteoblastogenesis and promotes adipogenesis. (A, B) Calvarial cells from *Runx1*^*f/f*^*Col1α1-Cre* (ff/Δ) and control (ff) newborn mice were applied to osteoblastogenesis assays (A) ALP and (B) Oil red staining. (C) Protein levels of Runx1, Cbfβ, Runx2, Atf4, Opn, and Osx were analyzed by Western blot analysis. (D) Quantification of western blot results in C. (E) *Runx1*^*f/f*^ *Col1α1-Cre*;GFP^-^ and GFP^+^ bone marrow MSCs were mixed together in different ratios and cultured in osteogenic medium. Adipocytes were labeled by Nile Red and couterstained by DAPI on Day 14. (F, G) Quantification of (F) Nile Red^+^GFP^-^/GFP^-^ and (G) Nile Red^+^GFP^+^/GFP^+^ ratios in E. Results are expressed as mean ± SD, n ≧ 3 in each group. *p < 0.05, **p < 0.01, ***p < 0.001.

*In vitro* osteoblastogenesis and adipogenesis were also examined in the *Runx1*-deficient cells. Calvarial cells from *Runx1*^*f/f*^*Col1α1-Cre* after 7 d of culture showed a significant reduction in the number of osteoblasts, as shown by reduced alkaline phosphatase (ALP) stain (Fig 3A). We also examined the ALP staining after 14 d of culture and found that *Runx1*^*f/f*^*Col1α1-Cre* cells exhibited significantly decreased bone formation (Fig 3A). Adipocyte formation was detected by Oil Red O staining, which demonstrated that a significantly higher number of *Runx1*^*f/f*^*Col1α1-Cre* cells were committed to the adipocyte lineage (Fig 3B). In addition, the ALP staining of *Runx1*^*f/f*^*Col2α1-Cre* calvarial cells osteogenesis induced for 14 days was also notably decreased (S2D Fig) while the Oil Red O staining showed the adipocytes accumulation was dramatically increased (S2E Fig) compared to its control. At the mRNA level, we found that expression levels of Runx1, Runx2, Atf4, Bmp7, Alk3, Ocn, Osx, and Col1a1 were significantly reduced in *Runx1*^*f/f*^*Col1α1-Cre* mice, while the expression of C/ebpa and Ppargγ (Pparg) were all significantly increased (S3 Fig). Furthermore, at the protein level, the levels of C/ebpa and Pparg were significantly increased in *Runx1*^*f/f*^*Col1α1-Cre* and *Runx1*^*f/f*^*Twist2-Cre* calvarial cells after osteogenesis induction for 14 days (S4 Fig). Western blot was used to analyze the protein levels of several key factors that influence osteoblast function in *Runx1*^*f/f*^*Col1α1-Cre* mice. *Runx1* deficiency reduced the expression of Runx2, Atf4, Opn, and Osx (at day 14), but not Cbfβ (Fig 3C and 3D). These data indicate that *Runx1* is critical for osteoblast differentiation and the lineage switch from osteoblasts to adipocytes by regulating the expression of critical downstream targets at the protein level. We next mixed GFP^−^; *Runx1*-deficient MSCs with GFP^+^ WT MSCs at different ratios and cultured them in osteogenic medium for 14 days to further examine whether *Runx1* antagonizes adipogenesis cell-autonomously. Adipocytes were stained with Nile Red and counterstained by DAPI. The co-culture experiment revealed that *Runx1*-deficient MSCs were more likely to differentiate into adipocytes: as the ratio of *Runx1*-deficient MSCs in the co-culture increased, more adipocytes were formed (Fig 3E and S5A–S5F Fig). These results demonstrated that co-culture with *Runx1*-deficient MSCs increased the adipogenesis rate of GFP+ normal cells (Nile Red^+^GFP^+^/GFP^+^ ratios 0.33%, 1.49%, 2.17%, and 4.05%, based on GFP^-^:GFP^+^ ratios 0:1, 1:1, 3:1, and 6:1, respectively) (Fig 3E–3G). A higher ratio of *Runx1*-deficient MSCs in the co-culture also increased adipogenesis in GFP^–^*Runx1* deficient cells (Nile Red^+^GFP^−^/GFP^−^ ratios 8.89%, 35.06%, and 61.53%, based on GFP^-^:GFP^+^ ratios 1:1, 3:1, 6:1, respectively) (Fig 3E and 3F). From the GFP^-^:GFP^+^ ratios of 3:1 to 6:1, there was a 1.75-fold increase in the adipocyte formation rate of GFP^–^*Runx1* deficient cells (Fig 3F). Adipocyte formation rate was increased by 5.97-, 16.15-, and 15.21-fold in *Runx1* deficient cells compared with WT GFP^+^ cells (Fig 3F and 3G) based on GFP^-^:GFP^+^ ratios 1:1, 3:1, and 6:1, respectively. In addition, the Oil-Red staining was dramatically increased in *Runx1*^*f/f*^*Col1α1-Cre* (S5G Fig) and *Runx1*^*ff*^*Twist2-cre* (S5H Fig) calvarial cells induced for 14 days with adipogenesis induction medium compared to their controls. These results indicate that *Runx1* regulates adipogenesis through both cell-autonomous and cell-non autonomous pathways.

### RNA-sequencing analysis showed that *Runx1* promotes osteogenesis and inhibits adipogenesis by orchestrating canonical BMP signaling, Non-canonical BMP/ERK signaling, and WNT signaling

**Fig 4:**
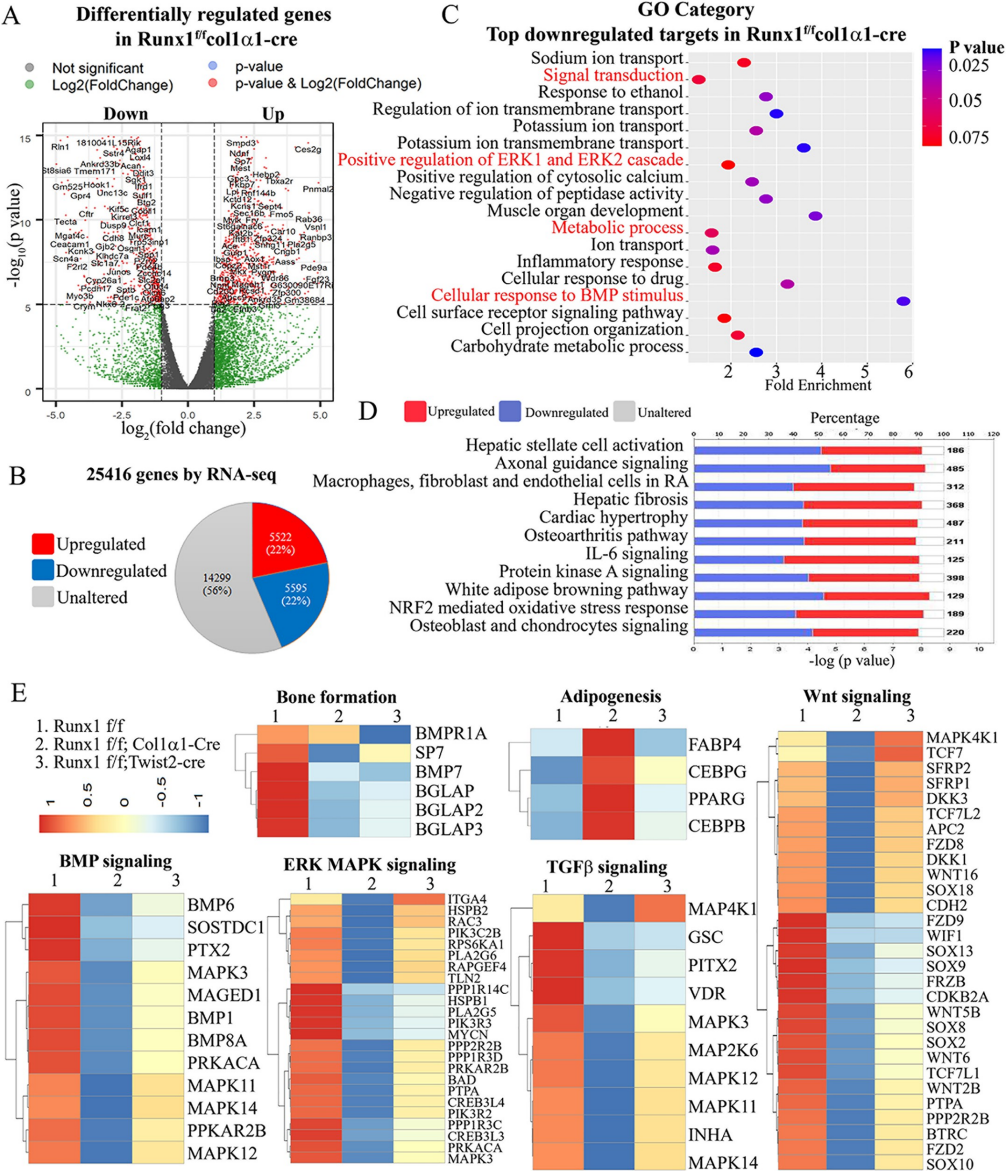
RNA-sequencing analysis of Runx1 CKO samples showed that Runx1 promotes osteogenesis and inhibits adipogenesis by orchestrating multiple signaling pathways involved in bone formation. (A) A volcano plot illustrating differentially regulated gene expression from RNA-seq analysis between the control and *Runx1*^*f/f*^*Col1α1-Cre* osteoblasts. Genes upregulated and downregulated are shown in red and blue, respectively. Values are presented as the log2 of tag counts. (B) RNA-seq comparison revealed a total of 25416 genes expressed, of which 5522 (21.7%) genes were upregulated and 5595 (22.3%) genes were downregulated. (C) Gene ontology (GO) functional clustering of genes that were downregulated for biological processes (The top most significantly affected categories are shown using KEGG analysis tool). (D) Ingenuity Pathway Analysis (IPA) showed the most significantly altered canonical pathways in *Runx1* deficient osteoblasts compared to its control. (E) Heatmaps of representative bone formation and adipogenesis-related genes in *Runx1*^*f/f*^*Col1α1-cre* and *Runx1*^*f/f*^*Twist2-cre mice* osteoblasts, and downregulated genes in BMP, ERK/MAPK, TGF-β and Wnt signaling in *Runx1*^*f/f*^*Col1α1-cre* and *Runx1*^*f/f*^*Twist2-cre* mice osteoblasts.

Using unbiased genome-wide RNA-seq data from *Runx1*^*f/f*^*Col1α1-Cre*, *Runx1*^*ff*^*Twist2-cre* and their control osteoblasts, we then examined *Runx1*-mediated transcriptional targets that could account for osteoblast differentiation defects and increased adipocytes. Among a total of 25416 differentially expressed genes (DEGs), transcripts of 5522 (21.7%) genes were upregulated, whereas transcripts of 5595 (22%) genes were downregulated in *Runx1*^*f/f*^*Col1α1-Cre* mice osteoblasts compared to the control osteoblasts (Fig 4A and 4B). In addition, gene ontology (GO) enrichment analysis demonstrated the top most significantly affected categories in genes that were downregulated in response to *Runx1* deficiency. Top GO downregulated categories were selected according to the P-values and enrichment score and illustrated as number of genes downregulated in respective category. Notably, among the top downregulated gene clusters were associated with *cellular response to BMP stimulus*, *positive regulation of ERK1 and ERK2 cascade*, *metabolic process*, and *signal transduction* (Fig 4C). We also utilized the Ingenuity Pathway Analysis (IPA) to examine the most significantly altered canonical pathways in *Runx1* osteoblasts which showed decreased osteoblast and chondrocyte signalling (Fig 4D). Heatmaps of representative bone formation and adipogenesis-related genes in *Runx1f/fCol1α1-Cre* and *Runx1*^*f/f*^*twist2-cre* showed that bone formation is downregulated while adipogenesis is upregulated by *Runx1* deficiency (Fig 4E), suggesting that *Runx1* is closely involved in bone formation homeostasis and may positively regulate osteoblast differentiation and negatively modulate adipocyte differentiation. We further demonstrated that the many genes in the Bmp, ERK/MAPK, TGF-beta and Wnt signalling pathways were significantly downregulated in *Runx1*^*f/f*^*Col1α1-Cre* and *Runx1*^*f/f*^*twist2-cre* mice osteoblasts compared to the control osteoblasts (Fig 4E). These results demonstrate that bone formation-related signalling pathway gene expression were significantly downregulated by *Runx1* deficiency and *Runx1* acts as a crucial regulator in osteoblasts homeostasis.

### RNA-sequencing analysis of Runx1 CKO samples revealed that Runx1 promotes chondrocyte and osteoblast development through regulating Ihh signalling

**Fig 5:**
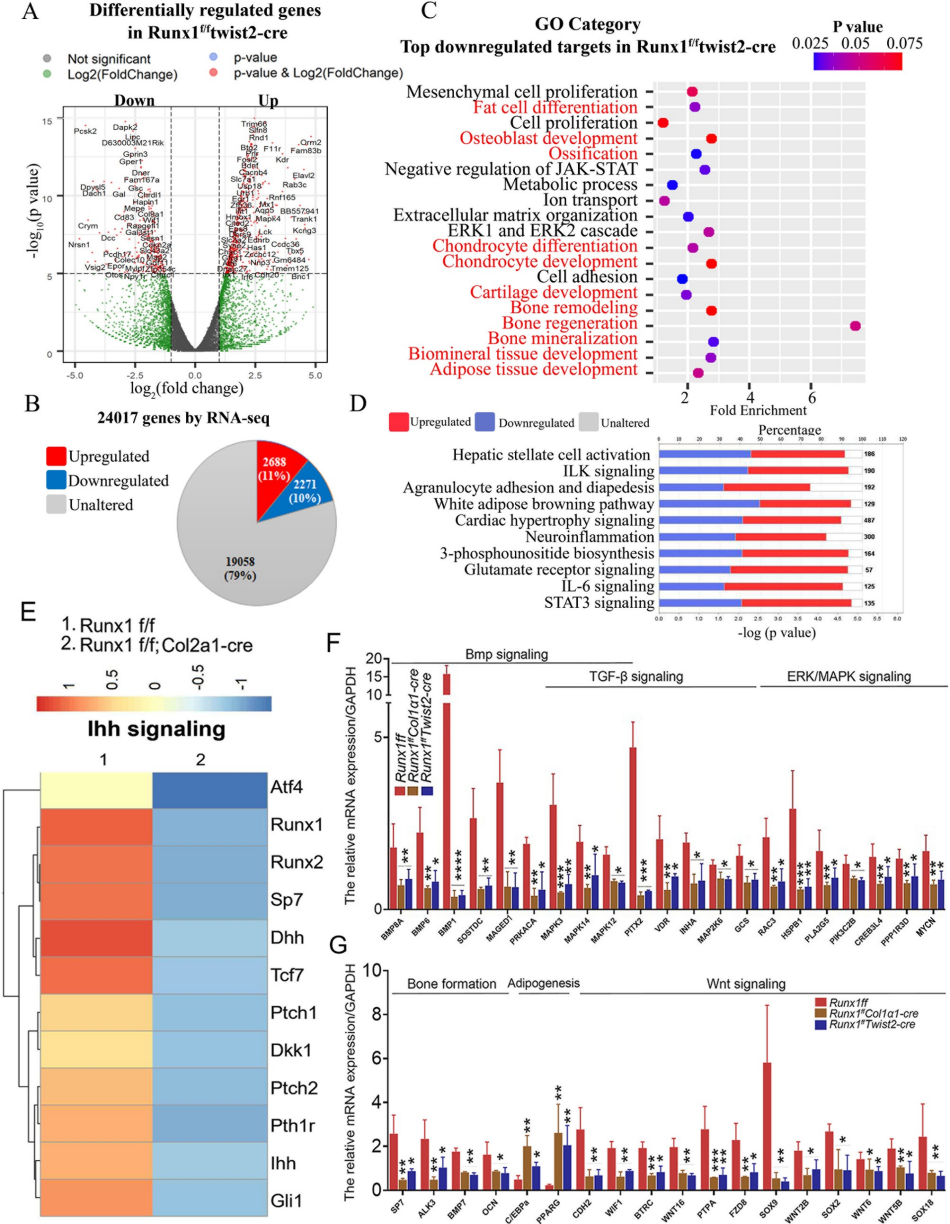
RNA-sequencing analysis of Runx1 CKO samples showed that Runx1 promotes chondrocyte and osteoblast development, and qPCR validation of RNA-sequencing analysis in *Runx1*^*f/f*^*Col1α1-Cre* newborn mice calvarial cells. (A) A volcano plot illustrating differentially regulated gene expression from RNA-seq analysis between the control and *Runx1*^*f/f*^*Twist2-Cre* osteoblasts. Genes upregulated and downregulated are shown in red and blue, respectively. Values are presented as the log2 of tag counts. (B) RNA-seq comparison revealed a total of 24017 genes expressed, of which 2688 (11%) genes were upregulated and 2271 (10%) genes were downregulated. (C) Gene ontology (GO) functional clustering of genes that were downregulated for biological processes (The top most significantly affected categories are shown using KEGG analysis tool). (D) Heatmaps of representative Ihh signaling genes in *Runx1*^*f/f*^*Col2α1-cre* mice cartilage. (E) qRT-PCR validation analysis shows the mRNA relative expression of Bmp, TGF-β and ERK/MAPK signaling-related genes in the control vs *Runx1*^*f/f*^*Col1α1-cre* and *Runx1*^*f/f*^*Twist2-cre* mice osteoblasts. (F) qRT-PCR validation analysis shows the mRNA relative expression of bone formation, adipogenesis and wnt signaling-related genes in the control vs *Runx1*^*f/f*^*Col1α1-cre* and *Runx1*^*f/f*^*Twist2-cre* mice osteoblasts. Results are expressed as mean ± SD, n = 4 in each group. *p < 0.05, **p < 0.01, ***p < 0.001, ***p < 0.0001.

Through unbiased genome-wide RNA-seq data from *Runx1*^*ff*^*Twist2-cre* and their control osteoblasts, we examined *Runx1*-mediated transcriptional targets that could account for osteoblast differentiation defects and increased adipocytes. Among the 24017 genes expressed, transcripts of 2688 (11%) genes were upregulated, whereas transcripts of 2271 (10%) genes were downregulated in *Runx1*^*f/f*^*Twist2-Cre* mice osteoblasts compared to the control osteoblasts (Fig 5A and 5B). In addition, gene ontology (GO) enrichment analysis demonstrated the top most significantly affected categories in genes that were downregulated as a result of *Runx1* deficiency. The top GO downregulated categories were selected according to the P-values and enrichment score and illustrated as number of genes downregulated in respective category. Consistent with our previous results, among the top downregulated gene clusters were associated with *Fat cell differentiation*, *osteoblast development*, *chondrocyte differentiation*, and *bone remodelling* (Fig 5C). Interestingly, per IPA results, inflammatory signaling pathways (ie. ILK, neuroinflammation, IL-6) were shown to be downregulated in *Runx1*^*f/f*^*Twist2-Cre* mice osteoblasts (Fig 5D). We next examined the expression of Ihh signalling pathway related genes in cartilage from *Runx1*^*f/f*^*Col2α1-Cre* mice which revealed that Ihh signalling is abrogated due to *Runx1* deficiency in cartilage (Fig 5E). This suggests that *Runx1* plays an important role in chondrogenesis, which is consistent with our recent study that revealed that *Runx1* up-regulates chondrocyte to osteoblast lineage commitment and promotes bone formation by enhancing both chondrogenesis and osteogenesis [32]. We then validated the differentially altered targets from Fig 4D and Fig 5D using qRT-PCR (Fig 5F and 5G). Consistent with the RNA-seq data, RT-qPCR confirmed the mRNA expression levels of the targets (Fig 5F and 5G). Collectively, these results showed that bone formation and chondrogenesis-related signalling pathway gene expression were significantly downregulated by *Runx1* deficiency and *Runx1* acts as a crucial regulator in osteoblasts and chondrocytes homeostasis.

### *Runx1* directly binds to the promoter regions of *Bmp7*, *Alk3*, and *Atf4* and upregulates their promoter activity

**Fig 6:**
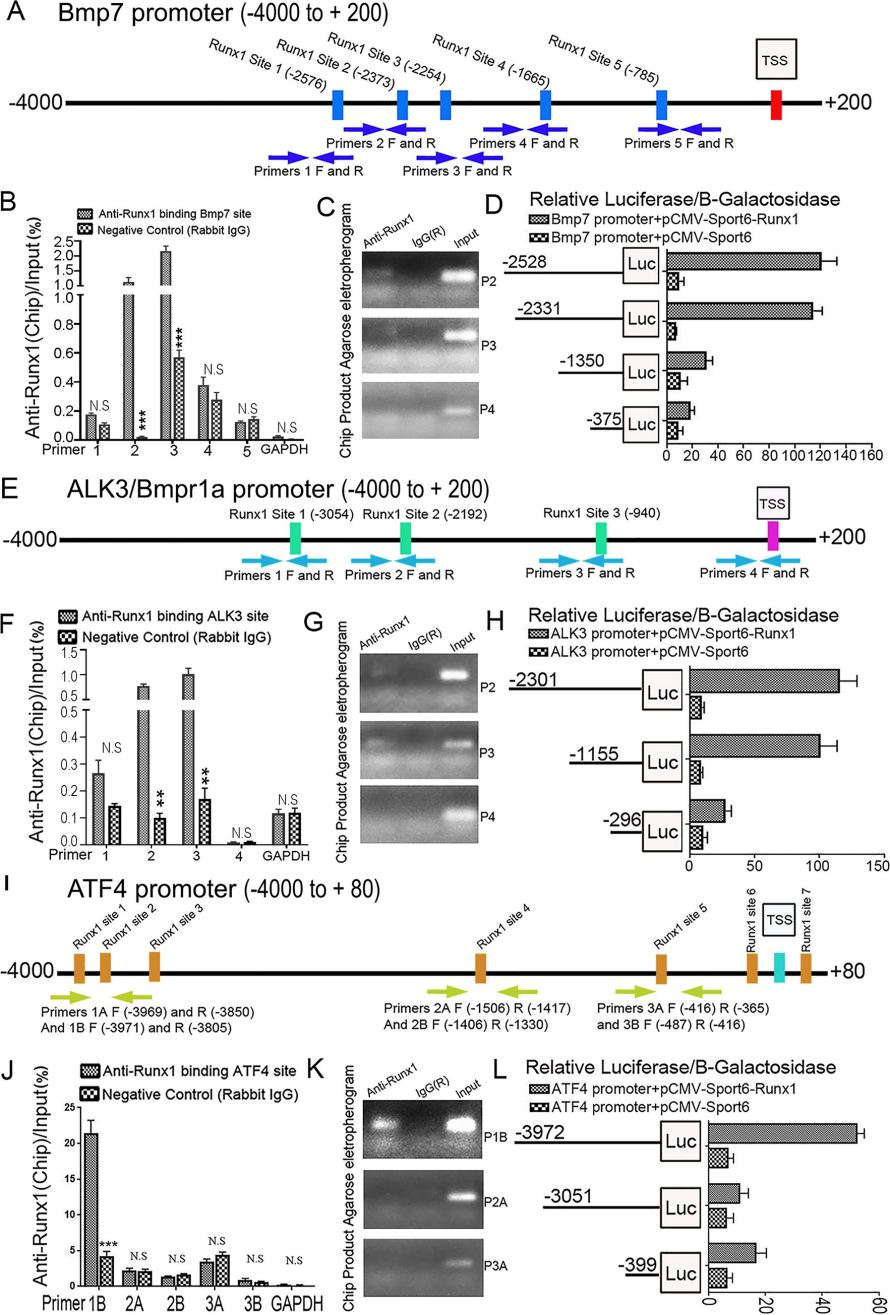
ChIP assay and promoter mapping revealed that *Runx1* directly binds to the promoter regions of *Bmp7*, *Alk3*, and *Atf4* and upregulates their promoter activity through the binding regions. (A) Schematic display of *Bmp7* (–4000/+200) promoter region: TSS, predicted *Runx1*-binding sites, and ChIP primers positions. (B) ChIP analysis of Runx1 binding to the *Bmp7* promoter in WT calvaria-derived osteoblasts using primers as indicated on the x-axis. Results are presented as ChIP/Input. (C) Agarose gel image using ChIP qPCR products in B. (D) *Bmp7* promoter fragments were inserted into pG13-basic vector. C3H10T1/2 cells were transfected with pG13-Bmp7–375 bp, –1350 bp, –2331 bp and –2528 bp. Luciferase was detected at 48 hours post transfection and normalized to β-gal activity. (E) Schematic display of *Alk3* (–4000/+200) promoter region: TSS, predicted *Runx1*-binding sites, and ChIP primers positions. (F) ChIP analysis of *Runx1* binding to the *Alk3* promoter in WT calvaria-derived osteoblasts using primers as indicated on the x-axis. Results are presented as ChIP/Input. (G) Agarose gel image using ChIP qPCR products in F. (H) *Alk3* promoter fragments were inserted into pG13-basic vector. C3H10T1/2 cells were transfected with pG13-Alk3–296 bp, –1155 bp and –2301 bp. Luciferase was detected at 48 hours post transfection and normalized to β-gal activity. (I) Schematic display of Atf4 (–4000/+80) promoter region: TSS, predicted *Runx1*-binding sites, and ChIP primers positions. (J) ChIP analysis of *Runx1* binding to the Atf4 promoter in WT calvaria-derived osteoblasts using primers as indicated on the x-axis. Results are presented as ChIP/Input. (K) Agarose gel image using ChIP qPCR products in J. (L) *Atf4* promoter fragments were inserted into pG13-basic vector. C3H10T1/2 cells were transfected with pG13-Atf4–399 bp, –3051 bp and –3972 bp. Luciferase was detected at 48 hours post transfection and normalized to β-gal activity. Results are presented as mean ± SD with n = 3. N.S, not significant, *p < 0.05, **p < 0.01, ***p < 0.001.

In order to further confirm that Bmp signaling pathway activation is downregulated by *Runx1* deficiency, we carried out chromatin immunoprecipitation (ChIP) assay to investigate evidence of Runx1 binding on *Bmp* genes promoters. As such, we found that *Runx1* may target *Bmp7* and its receptor *Alk3*. Interestingly, there are several *Runx1* binding sites in the *Bmp7* promoter region (-4000/+200) (Fig 6A). Given the highest ChIP input percentage value, *Runx1* potentially binds to binding site 2 and 3 in Bmp7 promoter (Fig 6B and 6C). The longest *Bmp7* promoter fragment (-2528/+80) resulted in the highest luciferase activity, which is significantly lower when driven by the other *Bmp7* promoter fragments (Fig 6D). Similarly, several *Runx1* binding sites were found in the *Alk3* promoter region (-4000/+200) (Fig 6E), with binding site 2 and 3 in the Alk3 promoter region are the most efficient locations for direct Runx1 interaction (Fig 6F and 6G). The promoter luciferase assay showed that luciferase activity was highest when driven by the longest *Alk3* promoter fragment (-2301/+80) (Fig 6H). In addition, we also found several predicted *Runx1* binding sites in the promoter (-4000/+200) of *Atf4* –an important transcription factor to regulate osteoblast differentiation (Fig 6I), potentially at binding site 1B (Fig 6J and 6K). Luciferase activity was highest when driven by the longest *Atf4* promoter fragment (-3972/+80) and was significantly lower when driven by the other *Atf4* promoter fragments (Fig 6L). In conclusion, our data demonstrated that *Runx1* binds to the promoter regions of *Bmp7*, *Alk3*, and *Atf4* to directly upregulate their promoter activity.

### Genetic dissection approach reveals Runx1/Bmp7/Alk3/Smad1/5/8/Runx2/ATF4 signaling pathway as the main mechanism that positively regulates bone formation to maintain postnatal and adult bone homeostasis

**Fig 7:**
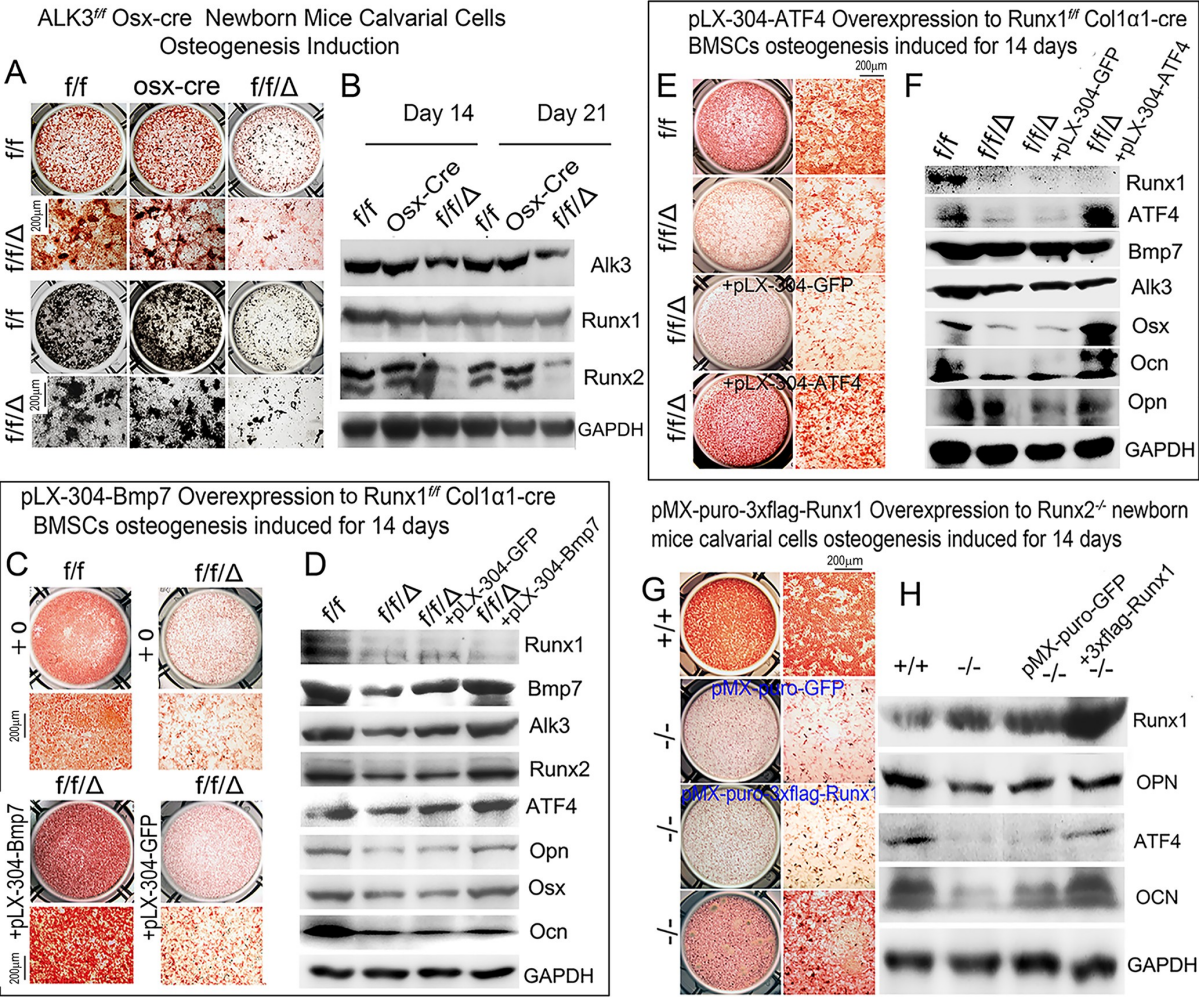
Calvarial cells from *Alk3*^*f/f*^*Osx-Cre* mice show impaired osteoblastogenesis and bone mineralization in vitro, and BMSCs from *Runx1*^*f/f*^*Col1α1-Cre* or Runx2^-/-^ mice show increased osteoblastogenesis and bone mineralization in vitro following overexpression of Atf4 or Runx1. (A) Calvarial cells from newborn *Alk3*^*f/f*^*Osx-Cre* (ff/Δ), wild-type (f/f), and Osx-cre mice were submitted to osteoblastogenesis assays. Osteoblast differentiation was analyzed by ALP activity on day 14 or by von Kossa staining on day 21. (B) Western blot was performed to detect protein levels of Alk3, Runx1, Runx2, Osx, and Ocn on days 14 and 21. GAPDH is shown as a control. (C) ALP staining following retrovirus-mediated overexpression of Bmp7 in day 14 BMSCs from Runx1f/fCol1α1-Cre mice. (D) Western blot was performed to detect protein levels of Runx1, Bmp7, Alk3, Runx2, Atf4, Opn, Osx, and Ocn on day 14. GAPDH is shown as a control. (E) retrovirus-mediated overexpression of Atf4 in BMSCs from newborn *Runx1*^*f/f*^*Col1α1-Cre* (ff/Δ) or wild-type (f/f) mice. Osteoblast differentiation was analyzed by ALP activity on day 14. (F) Western blot was performed to detect protein levels of Runx1, Atf4, Bmp7, Alk3, Osx, Ocn, and Opn on day 14. GAPDH is shown as a control. (G) retrovirus-mediated overexpression of Runx1 in calvarial cells from newborn *Runx2-/-* or wild-type (+/+) mice. Osteoblast differentiation was analyzed by ALP activity on day 14. (H) Western blot was performed to detect protein levels of Runx2, Runx1, Opn, Atf4, Osx, and Ocn on day 14. GAPDH is shown as a control. Results are expressed as mean ± SD, n≧3 in each group. N.S, not significant *p < 0.05, **p < 0.01, ***p < 0.001.

To understand how *Alk3* deficiency alters osteoblastogenesis, we examined calvarial cells from *Alk3*^*f/f*^*Osx-Cre* after 14d of culture which showed reduced alkaline phosphatase (ALP) (Fig 7A), indicating a decreased number of osteoblasts in the mutant cells. The reduction in mineralization observed in *Alk3*^*f/f*^*Osx-Cre* calvarial cells at day 21 was characterized by Von Kossa staining (Fig 7A). Western blot was used to analyze the expression of several important osteoblast function genes in *Alk3*^*f/f*^*Osx-Cre* mice. *Alk3* deficiency downregulated Runx2 expression, but not Runx1 (at day 14) (Fig 7B and S6A Fig). These findings indicate that *Alk3* deficiency alters the expression of critical downstream genes at the protein level which influences osteoblast differentiation. Using a pLX-304 vector, we generated a retrovirus encoding the GFP control and target gene Bmp7 cDNA to infect *Runx1*^*f/f*^*Col1α1-Cre* BMSCs after osteogenesis induction for 14 days, and we showed that Bmp7 was highly expressed post-infection (Fig 7C and S6B Fig), confirming that this retroviral system can sustain high gene expression for our overexpression studies. Retrovirus-mediated overexpression of Bmp7 in *Runx1*^*f/f*^*Col1α1-Cre* mutant mice BMSCs significantly rescued the decreased ALP staining compared to the pLX-304-GFP control (Fig 7C), and protein expression levels of Alk3, Runx2, Atf4, Opn, Osx, and Ocn (Fig 7D and S6B Fig), while the protein levels of Runx1 could not be rescued by Bmp7 overexpression (Fig 7D and S6B Fig). Similarly, retrovirus-mediated overexpression of Atf4 greatly rescued the ALP staining in *Runx1*^*f/f*^*Col1α1-Cre* mutant mice BMSCs (Fig 7E), as well as the protein levels of Atf4, Osx, Ocn, and Opn (Fig 7F and S6C Fig), while the protein levels of Runx1, Bmp7, and Alk3 could not be rescued by Atf4 overexpression (Fig 7F and S6C Fig). Runx1 overexpression in *Runx2*^*-/-*^ newborn mice (PCR result, S6D Fig) calvarial cells induced by osteogenesis medium rescued the ALP staining (Fig 7G), indicating that Runx1 can compensate for loss of Runx2 expression. In addition, the decreased protein levels of Atf4, Opn, and Ocn, which are genes critical to osteoblast differentiation, were all partially rescued (Fig 7H and S6E Fig).

### Overexpression of Runx1 increases osteoblasts and decreases adipocytes and osteoblast markers levels *in vitro*

**Fig 8:**
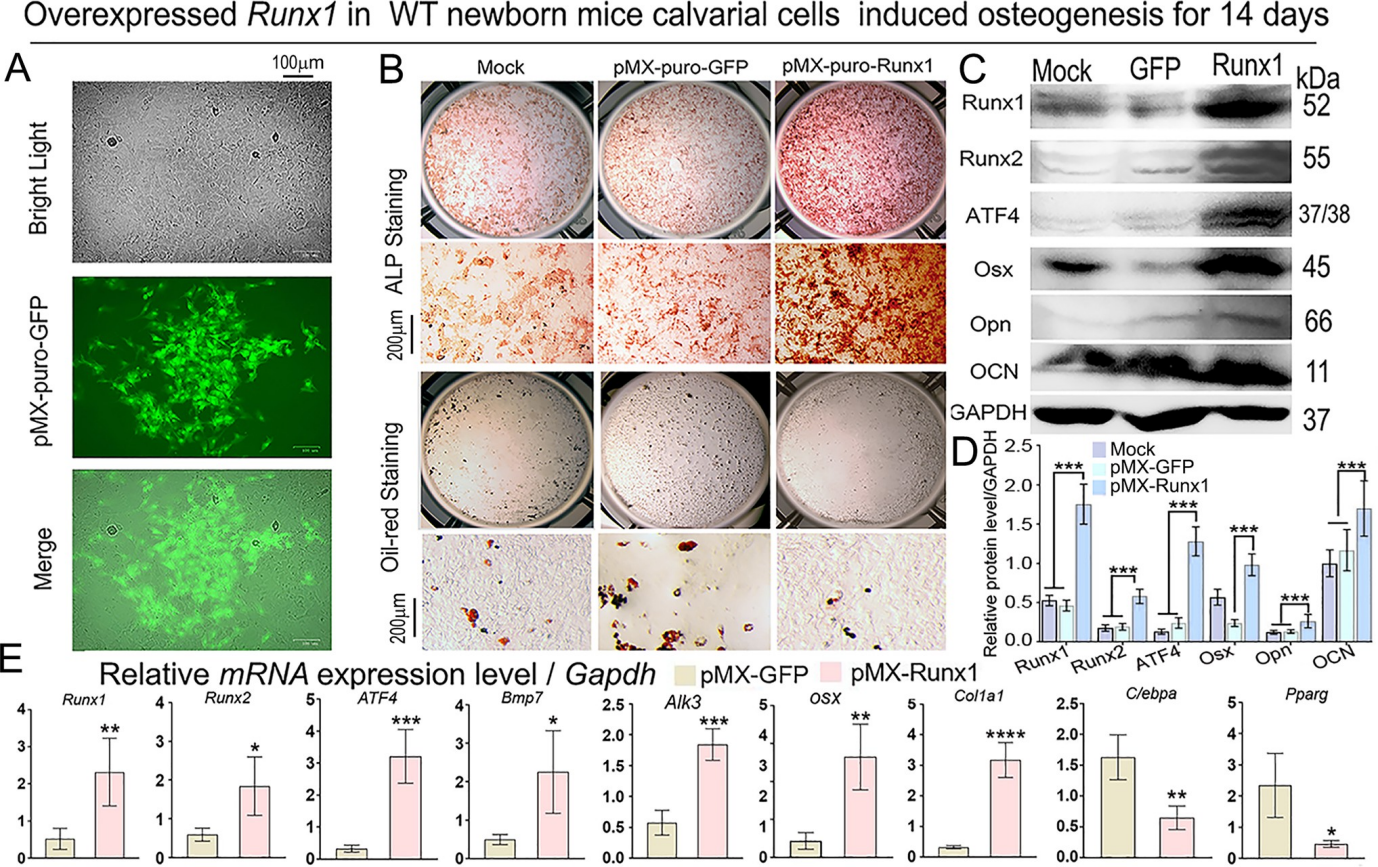
Overexpression of Runx1 increases osteoblasts and decreases adipocytes and osteoblast markers levels *in vitro*. (A) Overexpression of GFP in 14 days induced calvarial cells by retrovirus. (B) Retrovirus-mediated overexpression of Runx1 in 14 days induced calvarial cells. Osteoblast differentiation was analyzed by ALP activity on day 14, while adipocytes were analyzed by Oil red staining on day 14. (C) Western blot was performed on day 14 to evaluate the protein levels of Runx1, Runx2, Atf4, Osx, Opn, and Ocn. GAPDH is shown as a control. (D) Quantification of protein levels from western blot in C. (E) q-PCR was conducted on day 14 to evaluate the mRNA levels of Runx1, Runx2, Atf4, Bmp7, Alk3, Osx, Col1α1, C/ebpα and Pparg. All data are presented as mean ± SD, n = 4, NS denotes not significant, *p < 0.05, **p < 0.01, ***p < 0.001, ****p < 0.0001.

We generated a retrovirus encoding the *Runx1 cDNA* to infect newborn mouse calvarial cells, and it showed that GFP was successfully transfected into the calvarial cells (Fig 8A). Retrovirus-mediated overexpression of Runx1 in WT newborn mouse calvarial cells induced by osteogenesis medium for 14 days significantly increased the number of osteoblasts, as shown by significantly increased ALP positive cells while oil red staining was notably decreased, indicating reduced adipocytes after Runx1 overexpression (Fig 8B). We further found that the protein levels of Runx2, Atf4, Osx Opn, and Ocn were dramatically increased (Fig 8C and 8D), and at the mRNA level the expressions of Runx2, Atf4, Osx, Col1a1, Bmp7 and Alk3 were significantly upregulated, but the expression of adipocytes markers of C/ebpa and Pparg were significantly decreased following Runx1 overexpression (Fig 8E). Collectively, the results indicate that *Runx1* positively regulates osteoblasts and negatively modulates adipocytes through regulating the Runx1/Bmp7/Alk3/Smad1/5/8/Runx2/ATF4 signaling pathway to maintain postnatal and adult bone homeostasis.

### Runx1 deficiency significantly impaired both BMP signaling and TGF-β signaling in *Runx1*^*f/f*^*CKO* mice femur and tibia and calvarial cells

**Fig 9:**
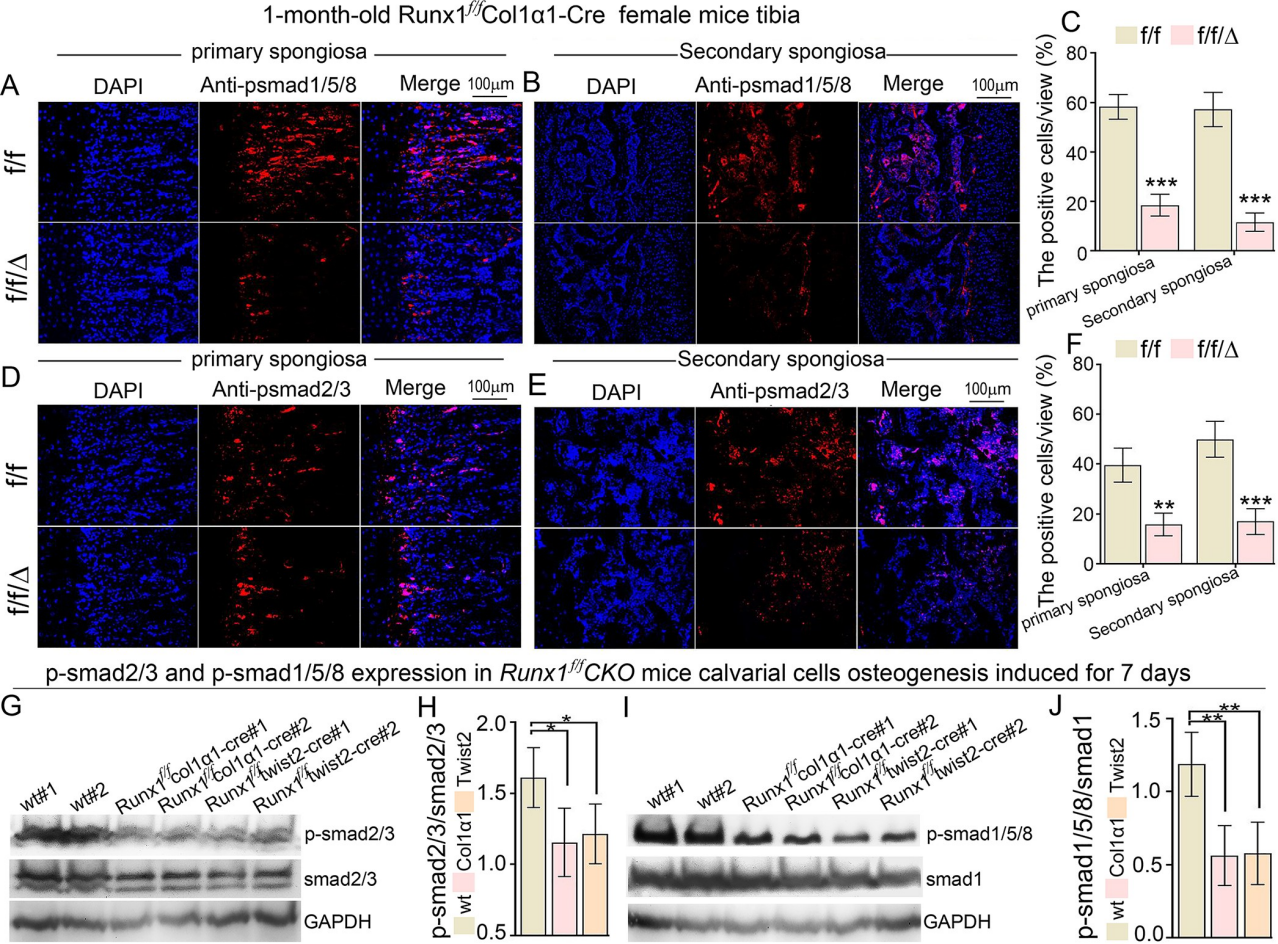
Reduced p-Smad1/5/8, and p-Smad2/3 expression indicated that impaired BMP signaling and TGF-β signaling in *Runx1*^*f/f*^*CKO* mice femur and tibia and calvarial cells. (A) Immunofluorescence staining of anti-phosphorylated Smad1/5/8 in *Runx1*^*f/f*^*Col1α1-Cre* 1-month-old female mice tibia primary spongiosa region (also known as trabecular bone) and (B) the secondary spongiosa region (secondary ossification center). (C) Quantification data for (A) and (B). (D) Immunofluorescence staining of anti-phosphorylated Smad2/3 in *Runx1*^*f/f*^*Col1α1-Cre* mice tibia primary spongiosa and (E) the secondary spongiosa. (F) Quantification data for (D) and (E). (G) p-smad2/3 protein expression in *Runx1*^*f/f*^*Col1α1-Cre* and *Runx1*^*f/f*^*twist2-Cre* newborn mice calvarial cells osteogenesis induced for 7 days. (H) Quantification data for (G). (I) p-smad1/5/8 protein expression in *Runx1*^*f/f*^*Col1α1-Cre* and *Runx1*^*f/f*^*twist2-Cre* newborn mice calvarial cells osteogenesis induced for 7 days. (J) Quantification data for (I). All data are presented as mean ± SD, n = 3 or 4, *p < 0.05, **p < 0.01, ***p < 0.001.

In order to further confirm whether the TGF-β/Bmp signaling was downregulated by *Runx1* deficiency. We performed immunofluorescence staining for a series of phosphorylated Smad genes that are downstream of TGF-β and Bmp signaling pathway in *Runx1* CKO mice femurs and tibias. In *Runx1*^*ff*^*Twist2-cre* newborn mice femur trabecular bone, the phosphorylation of Smad1/5/8 (p-Smad1/5/9) which were the canonical downstream of Bmp signaling pathway were significantly decreased as compared to its control (S7A and S7B Fig). In addition, the phosphorylation of Smad2/3 (p-Smad2/3) which was the canonical downstream of TGF-β signaling pathway was also reduced compared to its control (S7C and S7D Fig). In *Runx1*^*f/f*^*Col1α1-Cre* 1-month-old mice tibia primary (Trabecular bone) and secondary spongiosa (secondary ossification centre), Phosphorylation of Smad1/5/8 (Fig 9A–9C), and phosphorylation of Smad2/3 (Fig 9D–9F) were all significantly decreased compared to its controls. Furthermore, the ratio of p-smad2/3 normalized to total smad2/3 (Fig 9G and 9H) and p-smad1/5/8 normalized to total Smad1 (Fig 9I and 9J) were significantly decreased in *Runx1*^*f/f*^*Col1α1-Cre* and *Runx1*^*ff*^*Twist2-cre* newborn mice calvarial cells osteogenesis induction for 7 days compared to their controls. Through the *TGF-β1* Elisa experiment in *Runx1* CKO mice serum, we found that the *TGF-β1* expression was dramatically decreased in *Runx1*^*f/f*^*Col1α1-Cre* male and female mice serum as well as *Runx1*^*ff*^*Twist2-cre* male mice serum (S1G Fig). These results demonstrate *Runx1* may positively regulate osteoblast differentiation through regulating Smad-dependent TGF-β/Bmp signaling pathway(s).

### *Runx1* deficiency impairs β-catenin signaling *in vivo* and *in vitro*

**Fig 10:**
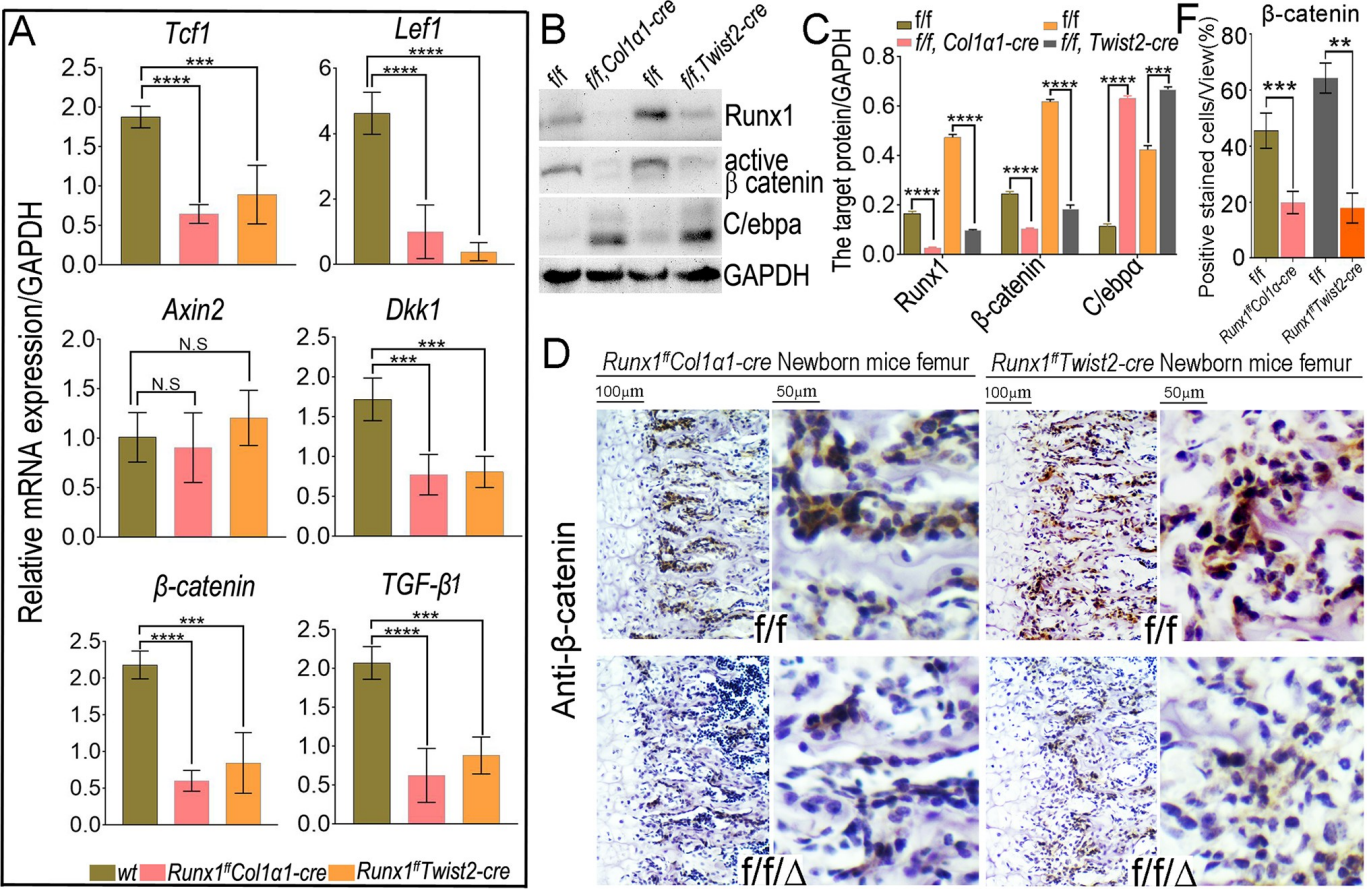
*Runx1* deficiency impairs β-catenin signaling *in vivo* and *in vitro*. (A) qRT-PCR analysis of *lef1*, *tcf1*, *axin2*, *dkk1*, *β-catenin* and *TGF-β* expression normalized by GAPDH. (B) Immunoblotting analysis of Runx1, β-catenin and C/ebpa protein level in both *Runx1*-deficient cells compared with their control cells. (C) The quantitative data analysis for the Runx1, β-catenin and C/ebpa protein levels normalized to GAPDH in B. (D) IHC staining of active β-catenin in *Runx1*-deficient mice femurs compared with their controls. (E) The quantitative data analysis for β-catenin level in the *Runx1*-deficient mice femurs compared with their controls. The data were presented as mean ± SD, n = 4. *p < 0.05, **p < 0.01, ***p < 0.001. NS, not significant.

In deciphering the molecular basis of *Runx1’s* roles in osteoblast−adipocyte lineage allocation, mRNA were harvested from WT, *Runx1*^*f/f*^*Col1α1-Cre*, and *Runx1*^*ff*^*Twist2-cre* calvarial cells, and further subjected to osteogenic medium for 14 d. The expression of genes that have been reported to be regulated by β-catenin (*lef1*, *tcf1*, *axin2*, *and dkk1*) were analyzed by quantitative reverse transcription PCR-qRT-PCR. The expression levels of *lef1*, *tcf1*, *and dkk1* were down-regulated significantly in both *Runx1*^*f/f*^*Col1α1-Cre* and *Runx1*^*ff*^*Twist2-cre* cells (Fig 10A). We also examined the expression of active β-catenin and C/ebpa in the calvarial cells and found a lower level of active β-catenin but a higher C/ebpa expression in *Runx1*-deficient osteoblasts (Fig 10B and 10C). In addition, we detected active β-catenin expression in the femurs of newborn *Runx1*^*f/f*^*Col1α1-Cre* and *Runx1*^*ff*^*Twist2-cre* compared to control mice femurs (Fig 10D and 10E). We found that the active β-catenin protein levels were down-regulated in the trabecular bone compared with that of the WT littermates. Taken together, our data indicate that β-catenin signaling was impaired in the absence of *Runx1*.

### *Runx1* overexpression rescued bone loss in OVX-induced osteoporosis

**Fig 11:**
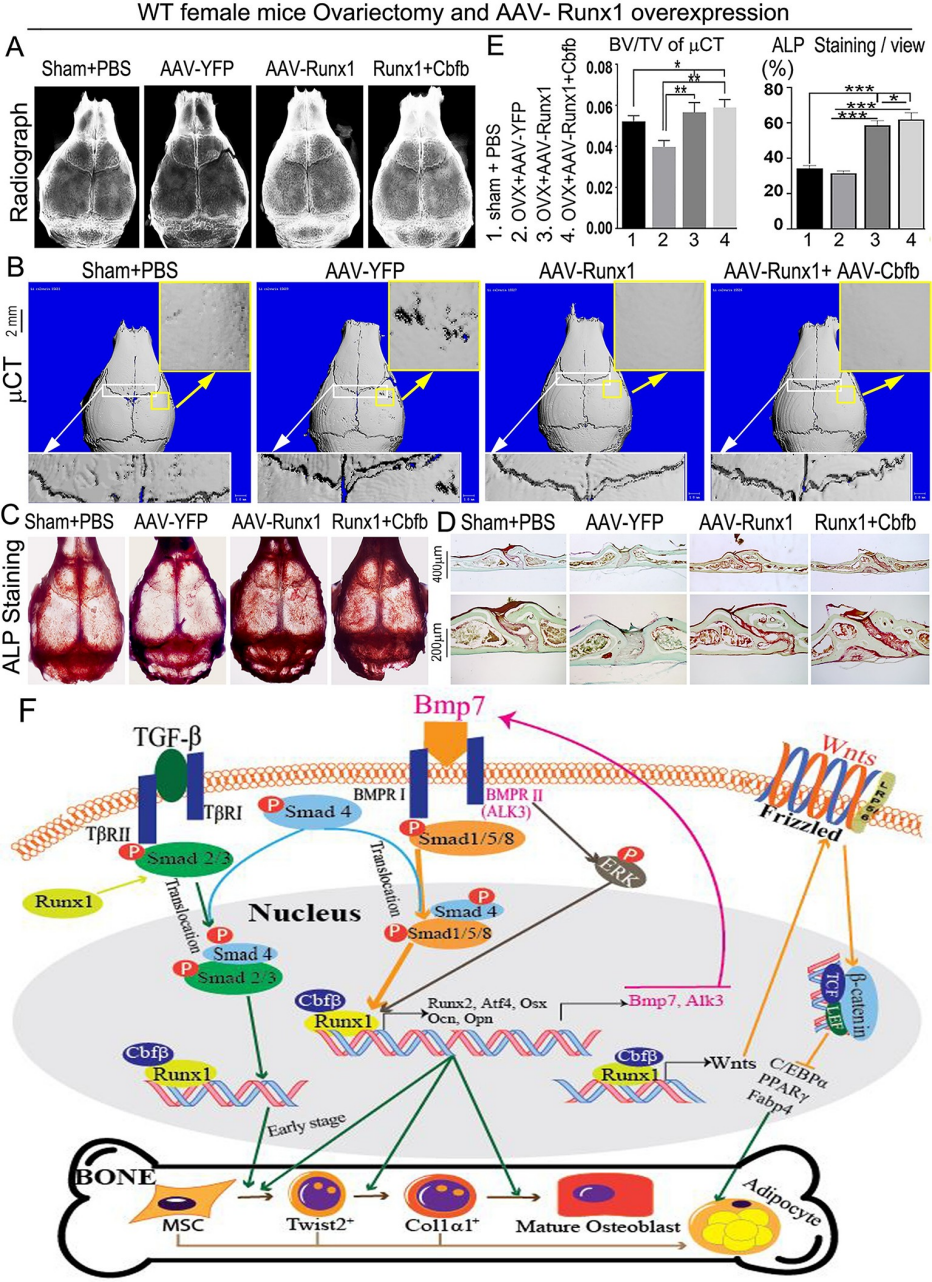
*Runx1* protects mice from OVX-induced bone loss and working model of *Runx1* regulating osteoblast-adipocyte lineage commitment. (A-D) Histological analysis of calvarial bones from 12-week-old female sham-operated mice (control), and ovarietomized (OVX) mice injected with AAV-YFP, AAV-Runx1, or AAV-Runx1+Cbfβ. (A) radiographic and (B) μ-CT images of skull calvaria. (C) Whole calvaria ALP staining. (D) ALP staining of calvaria frozen sections. (E) Quantification of bone volume per tissue volume (BV/TV) in (B), and quantification of ALP^+^ staining in (C). (F) Runx1 working model in enhancing bone formation and inhibiting adipogenesis at both early and late stages of osteoblast differentiation through orchestrating multiples signaling pathways. All data are presented as mean ± SD, n = 4, *p < 0.05, **p < 0.01, ***p < 0.001.

To investigate the therapeutic potential of *Runx1*, we overexpressed *Runx1* locally in mice using adeno-associated virus (AAV)-mediated gene overexpression to investigate its effects in protecting against bone degradation. To test the efficacy of AAV-Runx1 to protect against bone loss, we utilized the ovariectomized (OVX) animal model for OVX-induced bone loss due to estrogen depletion (Fig 11A–11C, sham+PBS group compared with OVX+YFP group). OVX mice were subjected to calvaria adjacent subcutaneous injection of AAV-YFP, AAV-Runx1, AAV-Cbfβ, or AAV-Runx1+Cbfβ. Radiographic and uCT analyses demonstrated that AAV-mediated overexpression of Runx1 with or without its binding partner, Cbfβ, significantly increased bone volume after estrogen depletion induced osteoporosis (Fig 11A, 11B and 11E). As assessed by ALP staining of whole calvaria and calvarial sections, AAV-Runx1 could rescue OVX-induced bone loss as shown by a significant increase in the number of osteoblasts (Fig 11C–11E) as compared with YFP group. These results demonstrated that *Runx1* may be an important therapeutic target to protect against pathological bone loss. In addition, working model map showed that *Runx1* positively regulates osteoblast differentiation by orchestrating multiple signaling pathways and inhibits adipogenesis (Fig 11F).

## Discussion

Taken together, our data demonstrate that *Runx1* is important to the maintenance of the osteoblast lineage and bone homeostasis by orchestrating multiple signaling pathways involved in bone formation, including the *Bmp7/Alk3/Smad1/5/8/Runx2/ATF4* and *WNT/Catenin* pathways (Fig 11F). Our data revealed that *Runx1* positively regulates both early and late stages of osteoblast lineage to promote bone formation and inhibit adipogenesis (Fig 11F). In contrast, *Runx2* is indispensable during bone development, but negatively regulates bone formation in late stages of osteoblast differentiation [25]. *Runx1* overexpression can rescue bone loss in OVX-induced osteoporosis (Fig 11A–11D).

### *Runx1* plays a critical role in up-regulating bone formation to maintain postnatal and adult bone homeostasis

*Runx1* is important for MSC lineage commitment to the early stages of chondrogenesis, as well as chondrocyte lineage commitment and differentiation [22, 24, 33]. Despite its high level of expression in osteoblasts, it is unclear whether Runx1 also plays a role in bone formation and bone homeostasis [15–17]. In previous studies, *Kimura et al*. deleted *Runx1* in mesenchyme cells by using *Prx1-cre*, and they observed only a slight and transient inhibition of sternal mineralization, while no obvious phenotype was found in the long bones [21]. However, in this study, our results demonstrated that *Runx1* conditional knockout mice using *Twist2-Cre* and *Col1α1-Cre* (*Runx1* CKO) displayed a severe osteoporosis phenotype (Fig 1 and S1 Fig). We noticed that both newborn *Twist2-Cre* and *Col1α1-Cre Runx1* mutant CKO mice exhibited with normal skeletal development. However, both *Twist2-Cre* and *Col1α1-Cre Runx1* mutant CKO mice displayed a severe osteoporosis phenotype, while a more severe osteoporosis phenotype was observed in 8-month-old mutant mice (Fig 1D). We also noticed that the osteoporosis phenotype in *Runx1*^*f/f*^*Col1α1-Cre* adult mutant CKO mice was more severe than that found in *Runx1*^*f/f*^*Twist2-Cre* adult CKO mice (Fig 1D). *Han et al* reported that Ctsk^+^ cells and Prx1^+^ cells in the periosteum might represent two subsets of mesenchymal stem cells with different anatomic distributions and functions [34], and only part of Prx1^+^ mesenchymal stem cells that are *Runx1*-deficient are in the periosteum of cortical bone. In contrast, all osteoblasts are *Col1α1*^+^ cells and all osteoblasts in *Runx1*^*f/f*^*Col1α1-Cre CKO* mice are *Runx1* deficient cells. Further, *Kimura et al*’s study focused on P0 to 3-week-old *Runx1*^*f/f*^*Prx1-cre* mice, but our research utilized P0 to 8-month-old *Runx1* osteoblast lineage conditional knockout (CKO) mice. This may explain why the previous *Runx1*^*f/f*^*Prx1-cre* mice model phenotypes were not obvious while our *Runx1*^*f/f*^*CKO* mouse models had striking phenotypes. To our knowledge, this is the first study of the role of *Runx1* during osteogenesis.

In RNA-seq analysis, we found that for bone formation related signaling pathway genes expression, the genes in the *Runx1*^*f/f*^*Col1α1* panel were significantly downregulated compared to the genes in the *Runx1*^*ff*^ *Twist2-cre* panel (Fig 4D), which demonstrates that *Runx1* may play a more important role in the mature osteoblast stage rather than in the osteoblast progenitor stage. This may also indicate that *Runx1*^*ff*^ Twist2-cre only knockout a part of mesenchymal stem cells that develop to osteoblasts. In this study, we found that *Runx1* deletion both at early or late osteoblast lineage stage leads to a severe osteoporotic phenotype with a significant increase in adipocytes (Figs 2 and 3). Notably, our recent studies demonstrated that Runx1 up-regulates chondrocyte to osteoblast lineage commitment and promotes bone formation by enhancing both chondrogenesis and osteogenesis by directly binding to the Ihh promoter to regulate its expression, indicating that *Runx1* directly regulates the transcriptional expression of chondrocyte genes [32]. Furthermore, Runx1 causes the expression of Runx2 and multiple bone-specific genes to increase, thereby mediating osteoblast differentiation and bone formation [35]. Our previous work has shown that Runx1 directly bind to *Runx2* and *Ocn* gene promoters, in which Runx2 expression was significantly upregulated [35].

### While *Runx2* is responsible for skeletal development and bone formation during embryonic development, *Runx1* may be responsible for up-regulating postnatal and adult bone formation

It has been demonstrated that Runx2 is essential for skeletal development [25, 36, 37], however, due to negative regulation of osteoblast maturation, overexpression of *Runx2* in cells of the osteoblastic lineage leads to osteopenia [29]. There is a long-standing question in bone biology to determine which transcription factors positively regulate postnatal to adult bone formation. We hypothesized that *Runx1* is a good candidate because *Runx1* is a member of the Runx protein family, highly expressed in pre-osteoblasts and osteoblasts, and forms a heterodimer with *Cbfβ*. We used loss-of-function and gain-of-function approaches, and genetics dissection approaches to demonstrate that *Runx1* functions as a positive regulator of postnatal to adult bone formation.

These results demonstrated that *Runx1* can partially replace *Runx2’s* function to positively regulate osteoblasts differentiation. Runx1 expression was detected in early and later stage skeletal cells, while Runx2 was less expressed in adult skeletons initially [30]. *Runx1* overexpression in *Runx2*^*-/-*^osteoblasts rescued expression of Atf4 and OCN, as well as bone formation *in vitro*, and compensated for loss of *Runx2* in *Runx2*^*-/-*^ osteoblasts. The deficiency of osteoblast activity in *Runx2*^*-/-*^ mice calvarial cells could be partially rescued after Runx1 was overexpressed in *Runx2*^*-/-*^ calvarial cells (Fig 7G). Our loss-of-function and gain-of-function of *Runx1* results demonstrate that *Runx1* positively regulates bone formation. These differences may illustrate that osteoblast differentiation related signaling pathways regulated by *Runx1* and *Runx2* are different, and *Runx1* regulates *Runx2* expression spatiotemporally. *Runx2* is important during bone development, but negatively regulates bone formation in late stages of osteoblast differentiation, while our results demonstrate that *Runx1* consistently positively regulates bone formation. We revealed that *Runx1* positively regulates both early and late stages of osteoblast lineage to promote bone formation and inhibit adipogenesis. This is consistent with our previous finding that Cbfβ, a Runx1 DNA-binding partner, governs osteoblast−adipocyte lineage commitment through enhancing β-catenin signaling and suppressing adipogenesis gene expression [38]. Here, we find that Runx1 plays an equally important role in the lineage switch from osteoblasts to adipocytes. Notably, our loss-of-function and gain-of-function approaches demonstrated that *Runx1* can positively regulate *Runx2* expression and compensate *Runx2* expression deficiency in bone formation (Fig 7H). Therefore, these results showed that *Runx1* can positively regulate osteoblasts differentiation through the Bmp7/Alk3/Runx2/Aft4 and WNT/Catenin signaling pathways as described in the proposed Runx1 working model (Fig 11F) to up-regulate postnatal and adult bone formation to maintain postnatal and adult bone homeostasis.

### Unbiased RNA-seq analysis and genetic dissection approach reveal Bmp7/Alk3/Smad1/5/8/Runx2/ATF4 signaling pathway as the main mechanism that positively regulates bone formation to maintain postnatal and adult bone homeostasis

Through RNA-seq analysis and qPCR validation, the top downregulated genes in *Runx1*-deficient cells were associated with *cellular response to BMP stimulus* and *positive regulation of ERK1 and ERK2 cascade* (Fig 4C), which demonstrated that Bmp and ERK signalling maybe involved in the regulation of osteoblast differentiation in the absence of *Runx1*. Through further analysis, we found that bone formation related signaling pathways such as Bmp, TGF-β, ERK/MAPK, and Wnt signaling pathways were all downregulated in osteoblast differentiation and bone formation by *Runx1* deficiency (Fig 4D). It has been reported that BMP actives ERK though the non-canonical pathway [39, 40].

We applied the ChIP and promoter activity mapping, loss-of-function and gain-of-function, and genetics dissection approaches on key regulators to characterize the mechanism underlying how Runx1 positively regulates osteogenesis and bone formation. *Runx1* plays an important role in postnatal bone homeostasis via directly binding to the *Bmp7*, *Alk3*, and *Atf4* promoters to activate Bmp7, Alk3, and Atf4 (Figs 6 and 7; S3 Fig). The osteogenesis activity and mineralization capacity were both significantly decreased in the *Alk3*^*f/f*^*Osx-cre* newborn mice calvarial cells (Fig 7A), as well as the expression of osteoblast gene *Runx2* (Fig 6B), which demonstrated that *Alk3* can positively regulate bone formation. Interestingly, the expression of Runx1 was not significantly changed while Runx2 was significantly decreased in *Alk3*^*f/f*^*Osx-cre* newborn mice calvarial cells (Fig 7B), which may illustrate that *Runx1* may be the upstream gene of Alk3, but *Alk3* can modulate *Runx2* expression during osteoblastogenesis. Notably, Bmp7 overexpression in *Runx1*^*ff*^*Col1α1*-cre mutant mice BMSCs elevates Alk3 expression and osteogenesis activity, and rescued the expression of a series of decreased osteoblast genes expression including ALP, Osx, Opn, Ocn (Fig 7C and 7D), as well as the expression of Runx2 and Atf4 in *Runx1* deficient osteoblasts (Fig 7D). Our data also demonstrated that *Runx1* can up-regulate expression of *Bmp7*, *Alk3* Runx2 and Atf4, but not vice versa (Fig 7). In addition, *Runx1* also promotes osteogenesis and inhibits adipogenesis in mice calvarial cell system (Fig 8B). BMP signaling up-regulates Smad1/5/8 activity, which is downstream of BMP signaling in the canonical Bmp signaling pathway [41].

We found that the reduced osteoblast activity in *Runx1*^*ff*^*Col1α1-cre* mutant mice BMSCs could be rescued by Atf4 overexpression, which also rescued the expression of osteoblast genes Osx, Ocn and Opn, while the decreased Runx1, Bmp7 and Alk3 expression could not be rescued (Fig 7F). This further demonstrated that *Atf4* could be regulated by *Runx1*, *Bmp7*, and *Alk3* but not vice versa. We then detected the phosphorylation level of Smad1/5/8 and found it was dramatically decreased in *Runx1*^*f/f*^*Twist2-Cre*, *Runx1*^*f/f*^*Col1α1-Cre* mice femurs, tibias, and calvarial cells was significantly decreased in *Runx1*^*f/f*^*Twist2-Cre* and *Runx1*^*f/f*^*Col1α1-Cre* mice osteoblasts (Fig 9–9H). We then sought to investigate whether *Runx2* and *Atf4* can regulate *Runx1* or *Bmp7/Alk3*. Collectively, these results demonstrated that *Runx1* positively regulates osteogenesis and bone formation via *Bmp7/Alk3/Smad1/5/8/Runx2/ATF4* signaling pathway as described in the proposed Runx1 working model (Fig 11F).

### *Runx1* promotes osteogenesis and inhibits adipogenesis by orchestrating multiple signaling pathways involved in bone formation to maintain postnatal and adult bone homeostasis

In the present study, we found Runx1 promotes osteogenesis and inhibits adipogenesis by mainly by orchestrating *Bmp7/Alk3/Smad1/5/8/Runx2/ATF4* signaling pathway at canonical BMP signaling pathway. However, RNA-sequencing analysis, Western blot, and qPCR validation of Runx1 CKO samples showed that the ERK/MAPK, TGF-beta and Wnt signalling pathways were also significantly downregulated in *Runx1* CKO mice (Fig 4), indicating the importance of multiple signalling pathways regulated by Runx1 in maintaining bone homeostasis. We hypothesized that while Runx1 promotes osteogenesis, adipogenesis also must be inhibited. Therefore, Runx1 orchestrates multiple signalling pathways involved in bone formation to maintain postnatal and adult bone homeostasis, i.e. Runx1 up-regulates the *Bmp7/Alk3/Smad1/5/8/Runx2/ATF4* signaling pathway via the canonical BMP signaling pathway and *Bmp7/Alk3/ERK* signaling pathway via the Non-canonical BMP signaling pathway to upregulate bone formation and maintain bone homeostasis. Meanwhile, Runx1 governs osteoblast−adipocyte lineage commitment through enhancing Wnt/β-catenin signaling and suppressing adipogenesis gene expression (Fig 4). We previously reported that Wnt10b/β-catenin signalling plays a key role in osteoblast-adipocyte cell lineage commitment [38]. Our results showed that Runx1 inhibits adipogenesis to maintain postnatal and adult bone homeostasis at different osteoblast differentiation stages through both cell-autonomous and cell-non autonomous pathways (Figs 1–3; S2 and S5 Figs). Given Runx1’s function in enhancing Wnt/β-catenin signaling and suppressing adipogenesis, Runx1 may form heterodimer with Cbfβ to inhibit adipogenesis gene expression as described in the proposed Runx1 working model (Fig 11F). Interstitially, the TGF-beta signalling pathway was also significantly downregulated in *Runx1* CKO mice, which is consistent with previous reports that TGF-beta regulates early osteoblast differentiation and inhibits Runx2 expression at later stages of osteoblast differentiation [42] as described in the proposed Runx1 working model (Fig 11F). TGF-β plays a role in the coupling of bone formation to bone resorption [42], thus the severe osteopetrosis phenotype observed in Runx1 CKO mice could be due to loss of TGF-β-mediated coupling of osteoblast-mediated bone formation and osteoclast-mediated bone resorption. Our data showed that Runx1 orchestrates BMP signaling and Wnt/β-catenin signaling pathway to promote bone formation and inhibit adipogenesis to maintain postnatal and adult bone homeostasis (Fig 11F).

### *Runx1* may function as an excellent target for the prevention of human osteoporosis

Osteoporosis, a disease resulting in bone weakening, is the most common cause of bone fractures among the elderly. Currently there are no effective drugs to treat osteoporosis. The mechanisms by which transcription factor(s) positively regulate postnatal bone formation remains unclear. *Runx2* is important during bone development, but negatively regulates bone formation in postnatal and adult mice. In this study, we used *Runx1*^*f/f*^*Col1α1-cre*, *Runx1*^*f/f*^*Col2α1-Cre* and *Runx1*^*f/f*^*Twist2-cre* of *Runx1 CKO* to define the function of *Runx1* in bone formation at different osteoblast differentiation stages. Our data showed that *Runx1* is indispensable to promotes osteogenesis and inhibits adipogenesis to maintain postnatal and adult bone homeostasis at different osteoblast differentiation stages (Figs 1–3; S2 and S5 Figs). It was reported that Runx2 overexpression transgenic mice exhibited severe osteopenia [43]. However, AAV mediated *Runx1* overexpression can rescue bone loss in OVX-induced osteoporosis through enhancing osteoblast proliferation and differentiation, which was further enhanced by co-overexpression with *Cbfβ*. Runx1 overexpression in *Runx2*^*-/-*^ newborn mice calvarial cells induced by osteogenesis medium rescued ALP staining, indicating that *Runx1* can compensate for loss of *Runx2* expression. Taken together, *Runx1* plays a central role in orchestrating multiple signaling pathways involved in bone formation and adipogenesis by orchestrating BMP, TGF-β, ERK/MAPK, and WNT signaling involved in bone formation as described in the proposed Runx1 working model (Fig 11F). However, how *Runx1* coordinates with these signaling pathways and how *Runx1* can prevent aging-related bone loss needs further exploration. *Runx1* may be a potential target of clinical significance in the development of novel treatment strategies for osteoporosis and other degenerative bone diseases.

Collectively, together with our previous findings and current additional finding, our study has elaborated the general picture of Runx1 action in skeleton and has provided new insight and advancement of knowledge in skeletal development. Specifically, this study focused on the role of Runx1 in different cell populations (i.e. mesenchymal cells and mature osteoblasts) with regards to BMP and Wnt signaling pathways and in the interacting network underlying bone homeostasis as well as adipogenesis, and has provided new insight and advancement of knowledge in skeletal development.

## Materials and methods

The study was approved by the University of Alabama at Birmingham (UAB) Animal Care and Use Committee, conformed to National Institutes of Health guidelines, and followed all recommendations of Animal Research: Reporting in Vivo Experiments (ARRIVE) guidelines. For more detailed description, please refer to **S1 Supplemental Materials**.

### Ethics statement

All animal experimentation was approved by the IACUC at the University of Alabama at Birmingham and was carried out according to the legal requirements of the Association for Assessment and Accreditation of the Laboratory Animal Care International and the University of Alabama at Birmingham Institutional Animal Care and Use Committee.

### Generation of Runx1^ff^CKO, Alk3^ff^Osx-cre, and Runx2^-/-^ Mice

All animal experimentation was carried out according to the legal requirements of the Association for Assessment and Accreditation of the Laboratory Animal Care International and the University of Alabama at Birmingham Institutional Animal Care and Use Committee. Jackson Laboratory, strain name B6.129P2- Runx1tm1Tani/J, JAX no. 008772 were crossed with skeletal tissue cell (MSCs and osteoblasts) using the *Twist2-Cre*, *Col1α1-Cre* (2.3 kb) and *Col2α1-Cre* mouse lines, respectively. *Twist2-cre* mouse line was from Jackson Laboratory, strain name, B6.129X1-Twist2tm1.1(cre)Dor/J, JAX no. 008712). *Col1α1-Cre* (2.3 kb) mouse line was kindly provided by Dr. Crombrugghe from the University of Texas, Houston, TX 77030. *Col2α1-Cre* mouse line was from Jackson Laboratory, strain name B6;SJL-Tg(Col2a1-cre)1Bhr/J, JAX no.003554. *Runx1*^*f/f*^ mice and *Alk3*^*f/f*^ [44] mice with tissue specific promoter-driven Cre were crossed to generate heterozygous mice, which were intercrossed to obtain homozygous CKO mice. *Runx2* heterozygous mice (*Runx2*^*+/-*^) intercrossed to obtain homozygous mice (*Runx2*^*-/-*^). All mice were maintained under a 12-h light–dark cycle with ad libitum access to regular food and water at the UAB Animal Facility. Both male and female mice of each strain were randomly selected into groups of five animals each. The investigators were not blinded during allocation, animal handling, and endpoint measurements. The study was approved by the UAB Animal Care and Use Committee, conformed to National Institutes of Health guidelines, and followed all recommendations of Animal Research: Reporting in Vivo Experiments guidelines.

### Histology and tissue preparation

Histology and tissue preparation were performed as described previously [45]. Murine femurs and tibiae were harvested, skinned, and eviscerated before fixing in 4% paraformaldehyde (PFA) in 1×PBS overnight. Samples were then dehydrated in ethanol and decalcified in 10% EDTA for 3 wk. For paraffin sections, samples were dehydrated in ethanol, cleared in xylene, embedded in paraffin, sectioned at 6μm with a Leica microtome, and then mounted on Superfrost Plus slides (Fisher).

### Cell culture and osteoblast function

Primary calvarial osteoblasts were isolated from newborn mice and BMSCs and seeded in culture at 3×10^3^ cells per square centimeter as described [15, 0].

### Serum P1NP, CTX-1 and TGF-β1 assay

3-month- old male and female mice serum was collected after 6-hour fasting, and the serum P1NP, CTX-1 and TGF-β1 activity was detected and quantified using the Human Pro-Collagen I alpha Duo Set ELISA (DY6220-05), Mouse Crosslaps (CTX-1) ELISA Kit (MBS724196) and TGF beta-1 Human/Mouse Elisa kit (50-174-92) according to the manufacturer’s instructions.

### RNA-sequencing analysis

Total mRNA was isolated using TRIzol reagent (Invitrogen Corp., Carlsbad, CA) from the osteoblasts that were cultured for 14 days in osteogenic differentiation medium following the manufacturer's protocol and was submitted to Admera Health (South Plainsfield, NJ) who assessed sample quality with the Agilent Bioanalyzer and prepared the library using the NEBnext Ultra RNA—Poly-A kit. Libraries were analyzed using Illumina next generation sequencing and relative quantification was provided by Admera Health.Read counts were subjected to paired differential expression analysis using the R package DESeq2. Volcano plot of differentially expressed genes was generated with R package Enhanced Volcano using log_2_ (fold change) and −log_10_ (p value) values. Genes were considered significant for upregulation/downregulation if *p* < 0.05. GO analysis was carried out using DAVID online tool (https://david.ncifcrf.gov/). Top GO downregulated categories were selected according to the *P*-values and enrichment score, and illustrated as number of genes downregulated in respective category. Signaling pathway data were analyzed through the use of IPA (QIAGEN Inc., https://www.qiagenbioinformatics.com/products/ingenuitypathway-analysis). We used the R package pheatmap to generate the differential gene expression heatmaps previously analyzed by IPA.

### Western blot analysis

Protein samples were prepared from calvaria-derived osteoblasts and BMSCs in protein lysis buffer as described [45, 46]. Proteins were resolved on SDS/PAGE and electrotransferred on nitrocellulose membranes.

### Adipogenesis assays

Confluent cultures of primary calvarial cells were subjected to adipogenic medium containing 0.1 μM dexamethasone, 50 μM indomethacin, and 5 μg/ml insulin for 14 days. The progression of adipogenesis was monitored under light microscope. At the end of culture period, cells were stained for lipid droplets using Oil Red-O stain as described.

### qRT-PCR analysis

Total RNA was isolated from cultured cells at day 7 and day 14 (as indicated) with TRIzol reagent (15596018; Life Technologies). Mouse cDNA was reverse-transcribed from 0.5 g total RNA with SuperScript VILO Master Mix (11755050; LifeTechnologies). The qRT-PCR was performed using the one step RT-PCR System as previously described [45, 46]. Primer sequences are presented in the S1 Table.

### Chromatin immunoprecipitation

Chromatin Immunoprecipitation (ChIP) was performed as described using primary osteoblast lysates [15]. After immunoprecipitation using rabbit polyclonal anti Runx1 antibody (ab23980; Abcam) and DNA extraction, quantitative PCR was performed using the primers in the promoter region of mouse Bmp7, Alk3 and Atf4 genes (primer sequences are presented in the S2 Table).

### Promoter luciferase assay

The promoter region (−) and (+) of the mouse Bmp7, Alk3 and Atf4 gene was amplified by PCR using Bmp7, Alk3 and Atf4 Bac clone (cat#CH29-27K23; CHORI). Primer sequences are available in S3 Table. Then the promoter regions were inserted into the pGL3-basic vector to construct the pGL3-Bmp7, Alk3 and Atf4 promoter vectors and respectively. The insertions of the constructs were confirmed by sequencing. C3H10T1/2 cells were cultured in 24-well plates, and were transiently transfected with a DNA mixture containing the pGL3- Bmp7, Alk3 and Atf4 construct respectively (0.3μg) and β-GAL-expressing plasmids (0.03 μg using Lipofectamine and Plus reagents. Luciferase was detected using Glo Luciferase Assay System (Promega) 48 h post transfection as described [15]. The β-GAL activity of the cell lysates was analyzed using β-Galactosidase Enzyme Assay System (E2000; Promega). The level of luciferase activity was normalized to the level of β-GAL activity.

### OVX-induced bone destruction and AAV-Runx1 and AAV-Cbfβ treatment

OVX or sham was performed on two-month-old female mice. One week later and two weeks later those mice were administered with a local calvarial injection of 30ul AAV (titer 10^9−10^/ml) expressing YFP, Runx1, or Cbfβ. Mice were harvested 5 weeks after OVX operation and fixed in 4% PFA. Calvaria bone were analyzed by X-ray, u-CT and whole-mount TRAP staining. Calvarial were also decalcified for 3 days, immersed in 30% sucrose overnight and then submitted to frozen section and ALP staining.

### Statistical analysis

The number of animals used in this study was determined in accordance with power analysis and our previous studies [45, 46]. In brief, our study used five mice per group per experiment. Data are presented as mean ± SD (n ≥ 3). Statistical significance was assessed using *Student’s t* test. Values were considered statistically significant at *P* < 0.05. Results are representative of at least three individual experiments. Figures are representative of the data.

## Supporting information

S1 Fig Bone density was decreased and bone formation was impaired in Runx1f/fCol1α1-Cre and Runx1f/fTwist2-Cre mice. (A) X-ray for 6-week-old male and female *Runx1*^*f/f*^*Col1α1-Cre* mutant and its control mice. (B) X-ray for 4-week-old male, 6-week-old female and 17-week-old male *Runx1*^*f/f*^*Twist2-Cre* mutant and its control mice. (C, D) Calcein double label and Mineral apposition rate of (C) *Runx1*^*f/f*^*Col1α1-Cre* and (D) *Runx1*^*f/f*^*Twist2-Cre* 3-month-old male mice. (E-G) ELISA to detect the levels of (E) P1NP, (F) CTX-1, and (G) TGF-β. All data are presented as mean ± SD, n = 4, *p < 0.05, **p < 0.01, ****p < 0.0001.
(TIF)

S2 Fig Bone formation was decreased while adipogenesis was increased in Runx1f/fCol2α1-Cre mice. (A) ALP staining of 3-month-old *Runx1*^*f/f*^*Col2α1-Cre* female mice femurs and its control (*f/f*). The red arrow refer to adipocyte. (B) H&E staining of 3-month-old *Runx1*^*f/f*^*Col2α1-Cre* male mice femurs and its control (*f/f*). The red arrow refer to adipocyte. (C) ALP staining of 6-month-old *Runx1*^*f/f*^*Col2α1-Cre* male mice femurs and its control (*f/f*). The red arrow refer to adipocyte. (D) ALP staining of *Runx1*^*f/f*^*Col2α1-Cre* newborn mice calvarial cells osteogenesis induction for 14 days compared to its control (*f/f*). (E) Oil-Red Staining for *Runx1*^*f/f*^*Col2α1-Cre* newborn mice calvarial cells osteogenesis induction for 14 days compared to its control (*f/f*).
(TIF)

S3 Fig qPCR result of osteogenesis and adipogenesis gene markers expression in Runx1f/fCol1α1-Cre newborn mice calvarial cells. *Runx1*, *Runx2*, *Atf4*, *Osx*, *Ocn*, *Col1α1*, *Bmp7*, *Alk3*, *C/ebpα*, *and Pparg* expression in *Runx1*^*f/f*^ and *Runx1*^*f/f*^*Col1α1-Cre* newborn mice osteoblasts induced for 14 days. All data are presented as mean ± SD, n = 4, *p < 0.05, **p < 0.01, ***p < 0.001.
(TIF)

S4 Fig Adipogenesis regulator C/ebpα and Pparg expression was upregulated in Runx1 deficient calvarial cells osteogenesis induction for 14 days. (A) Anti-Runx1, C/ebpα and Pparg expression in *Runx1*^*f/f*^*Col1α1-cre* and *Runx1*^*f/f*^*Twist2-cre* calvarial cells osteogenesis induction for 14 days. (B) Quantification data of (A). All data are presented as mean ± SD, n = 3, ** *p* < 0.01, ***p < 0.001.
(TIF)

S5 Fig Co-culture of Runx1f/fCol2α1-Cre and Runx1f/fTwist2-Cre and wild-type cells demonstrated that Runx1 regulates osteoblast−adipocyte lineage commitment both cell-autonomously and non-autonomously at various differentiation stage. (A-C) *Runx1*^*f/f*^*Col2α1-Cre*; GFP^-^ and GFP^+^ bone marrow MSCs were mixed together in different ratios and cultured in osteogenic medium. (A) Adipocytes were labeled by Nile Red and couterstained by DAPI on Day 14. Quantification of (B) Nile Red^+^GFP^-^/GFP^-^ and (C) Nile Red^+^GFP^+^/GFP^+^ ratios in A. (D-F) *Runx1*^*f/f*^*Twist2-Cre*;GFP^-^ and GFP^+^ bone marrow MSCs were mixed together in different ratios and cultured in osteogenic medium. (D) Adipocytes were labeled by Nile Red and couterstained by DAPI on Day 14. Quantification of (E) Nile Red^+^GFP^-^/GFP^-^ and (F) Nile Red^+^GFP^+^/GFP^+^ ratios in D. (G) Oil-Red staining of *Runx1*^*f/f*^*Col2α1-Cre* and (H) *Runx1f/fTwist2-Cre* calvarial cells adipogenesis induction medium for 14 days. The data were presented as mean ± SD, n = 8. *p < 0.05, **p < 0.01.
(TIF)

S6 Fig Calvarial cells from Alk3f/fOsx-Cre mice show impaired osteoblastogenesis and bone mineralization in vitro, and BMSCs from Runx1f/fCol1α1-Cre or calvarial cells from Runx2-/- mice show increased osteoblastogenesis and bone mineralization in vitro following overexpression of Atf4 or Runx1. (A) Quantification of western blot data in Fig 5B. (B) Quantification of western blot data in Fig 5D. (C) Quantification of western blot data in Fig 5F. (D) PCR was used to determine Runx2 alleles (f/f, f/+, +/+, or deletion). (E) Quantification of western blot data in Fig 5H. All data are presented as mean ± SD, n = 3, N.S denotes not significant. *p < 0.05, **p < 0.01, ***p < 0.001.
(TIF)

S7 Fig Impaired p-Smad1/5/8 and p-Smad2/3 expression in Runx1f/fTwist2-Cre newborn mice femur. (A) Immunofluorescence staining of anti-phosphorylated Smad1/5/8 in *Runx1*^*f/f*^*Twist2-Cre* newborn mice femur compared to its control. (B) Quantification data of (A). (C) Staining of anti-phosphorylated Smad2/3 in *Runx1*^*f/f*^*Twist2-Cre* newborn mice femur compared to its control. (D) Quantification data of (C). All data are presented as mean ± SD, n = 3, *p < 0.05, ***p < 0.001.
(TIF)

S1 Table Primers used for qPCR. (XLSX)

S2 Table Primers used for ChIP assay. (XLSX)

S3 Table Primers used for subcloning. (XLSX)

S1 Text Supplemental Materials and Methods. (DOC)
