# Correction: Runx1 is a central regulator of osteogenesis for bone homeostasis by orchestrating BMP and WNT signaling pathways

**DOI:** 10.1371/journal.pgen.1010480

**Published:** 2022-10-31

**Authors:** Chen-Yi Tang, Mengrui Wu, Dongfeng Zhao, Diep Edwards, Abigail McVicar, Yuan Luo, Guochun Zhu, Yongjun Wang, Hou-De Zhou, Wei Chen, Yi-Ping Li

In order to facilitate enhanced reproducibility, the authors have completed the public deposition of all RNA-seq data to Gene Expression Omnibus (GEO) under accession Series GSE213930 (https://www.ncbi.nlm.nih.gov/geo/query/acc.cgi?acc=GSE213930).
