# Supplementary figures and images for: Runx1 is a central regulator of osteogenesis for bone homeostasis by orchestrating BMP and WNT signaling pathways
Source: PLoS Genet. 2021 Jan 21;17(1):e1009233. doi: 10.1371/journal.pgen.1009233 (PMC7819607; doi:10.1371/journal.pgen.1009233)

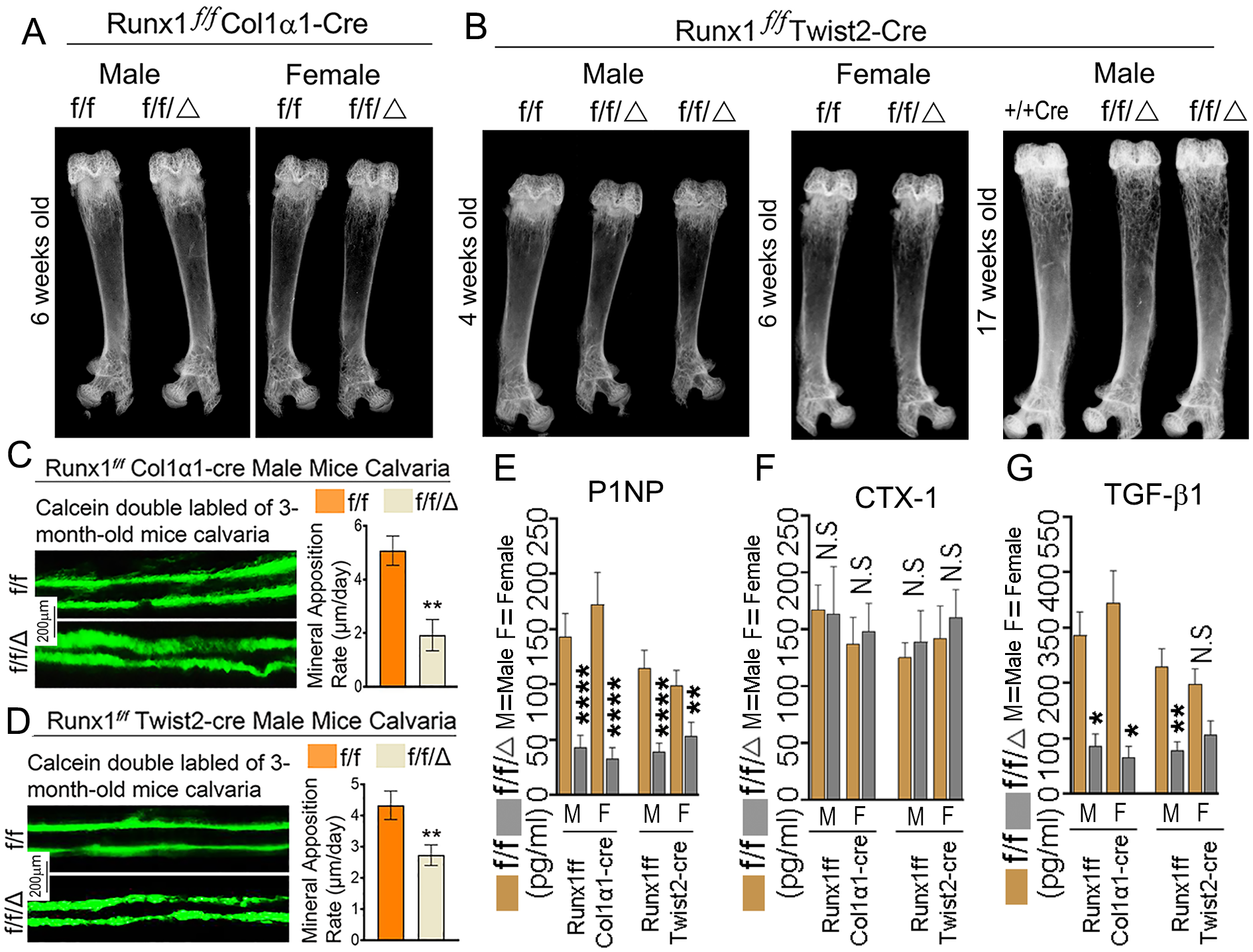

Supplement: S1 Fig — (A) X-ray for 6-week-old male and female Runx1f/fCol1α1-Cre mutant and its control mice. (B) X-ray for 4-week-old male, 6-week-old female and 17-week-old male Runx1f/fTwist2-Cre mutant and its control mice. (C, D) Calcein double label and Mineral apposition rate of (C) Runx1f/fCol1α1-Cre and (D) Runx1f/fTwist2-Cre 3-month-old male mice. (E-G) ELISA to detect the levels of (E) P1NP, (F) CTX-1, and (G) TGF-β. All data are presented as mean ± SD, n = 4, *p < 0.05, **p < 0.01, ****p < 0.0001. (TIF) [file pgen.1009233.s001.tif]

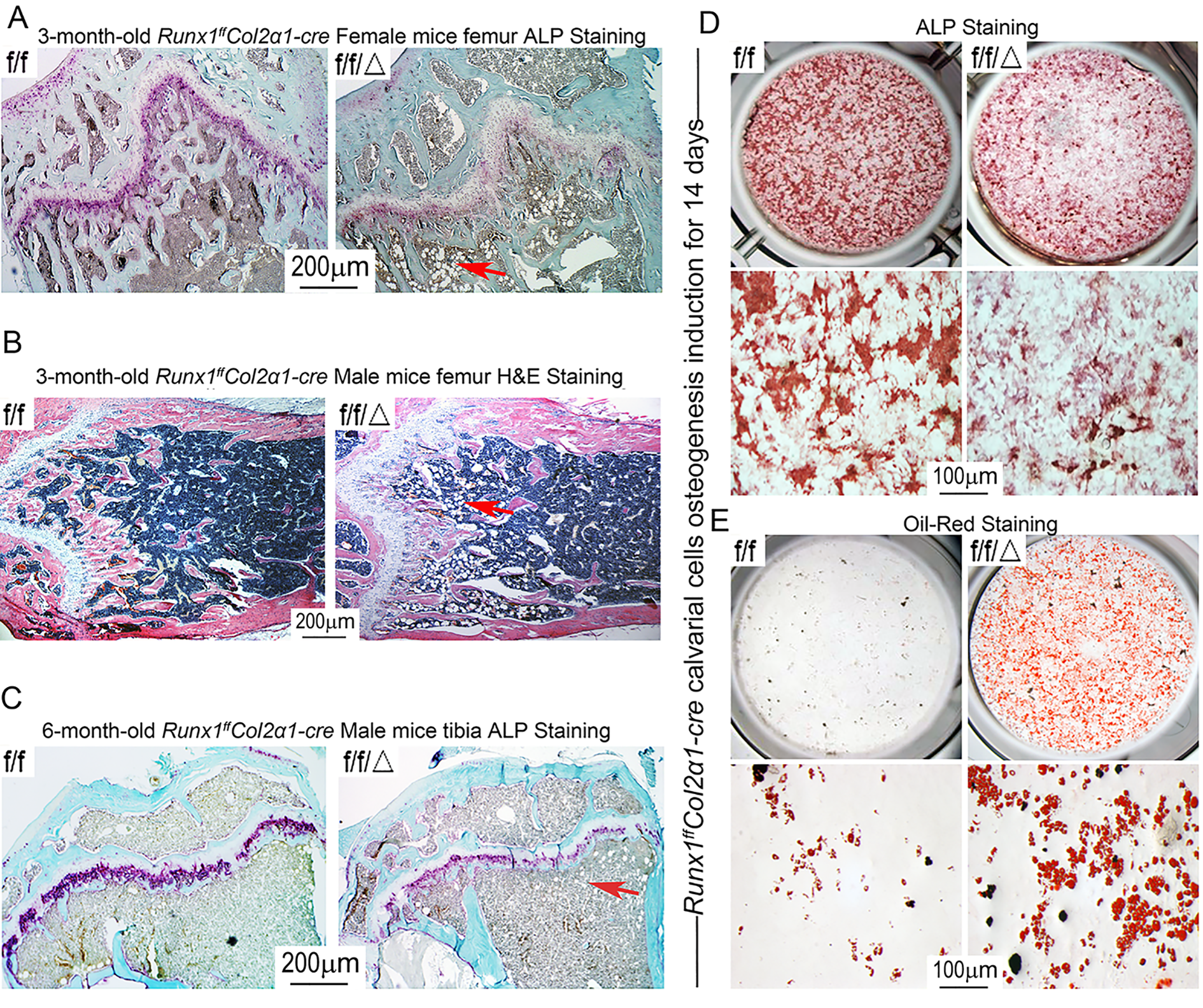

Supplement: S2 Fig — (A) ALP staining of 3-month-old Runx1f/fCol2α1-Cre female mice femurs and its control (f/f). The red arrow refer to adipocyte. (B) H&E staining of 3-month-old Runx1f/fCol2α1-Cre male mice femurs and its control (f/f). The red arrow refer to adipocyte. (C) ALP staining of 6-month-old Runx1f/fCol2α1-Cre male mice femurs and its control (f/f). The red arrow refer to adipocyte. (D) ALP staining of Runx1f/fCol2α1-Cre newborn mice calvarial cells osteogenesis induction for 14 days compared to its control (f/f). (E) Oil-Red Staining for Runx1f/fCol2α1-Cre newborn mice calvarial cells osteogenesis induction for 14 days compared to its control (f/f). (TIF) [file pgen.1009233.s002.tif]

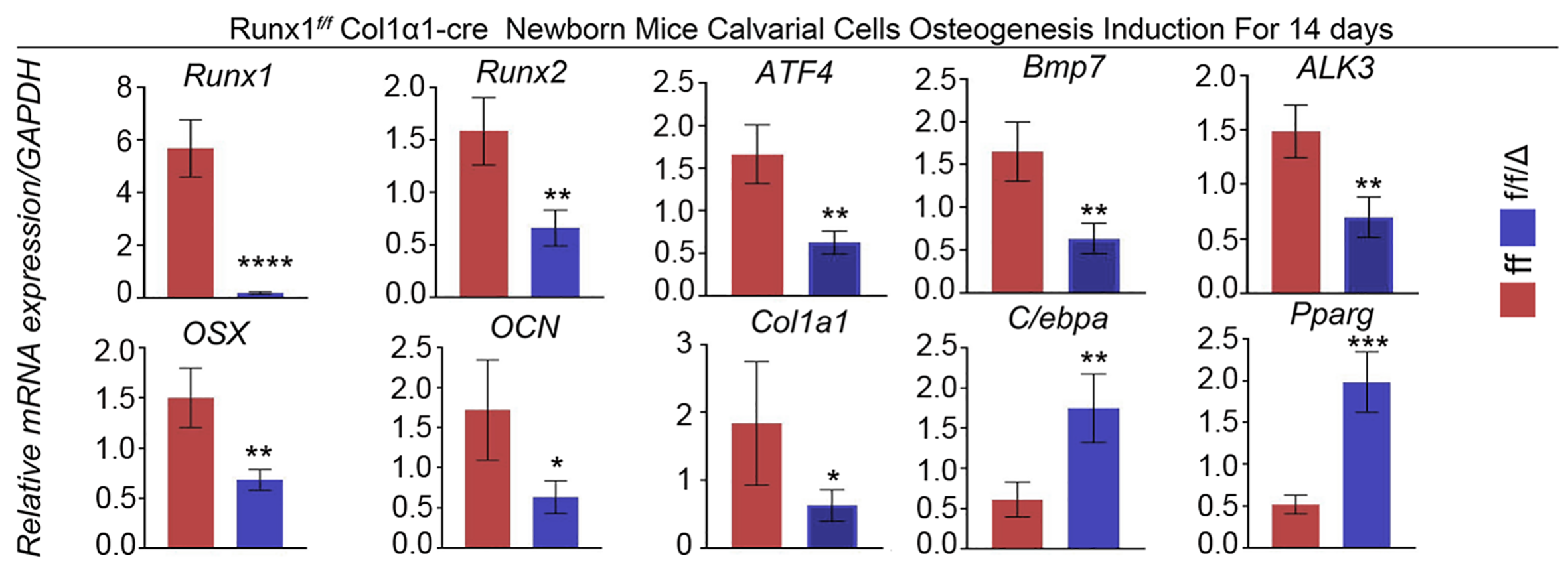

Supplement: S3 Fig — Runx1, Runx2, Atf4, Osx, Ocn, Col1α1, Bmp7, Alk3, C/ebpα, and Pparg expression in Runx1f/f and Runx1f/fCol1α1-Cre newborn mice osteoblasts induced for 14 days. All data are presented as mean ± SD, n = 4, *p < 0.05, **p < 0.01, ***p < 0.001. (TIF) [file pgen.1009233.s003.tif]

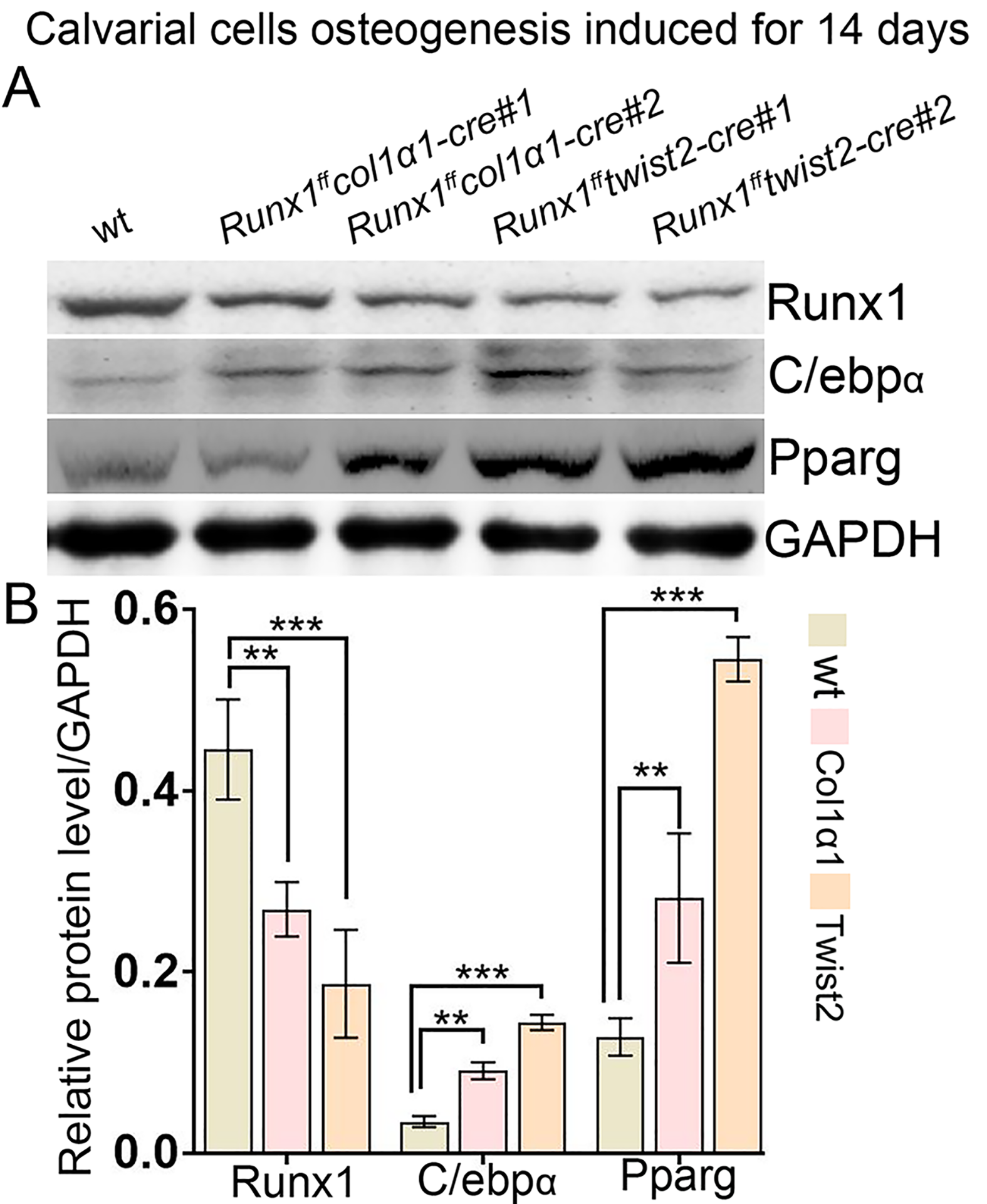

Supplement: S4 Fig — (A) Anti-Runx1, C/ebpα and Pparg expression in Runx1f/fCol1α1-cre and Runx1f/fTwist2-cre calvarial cells osteogenesis induction for 14 days. (B) Quantification data of (A). All data are presented as mean ± SD, n = 3, ** p < 0.01, ***p < 0.001. (TIF) [file pgen.1009233.s004.tif]

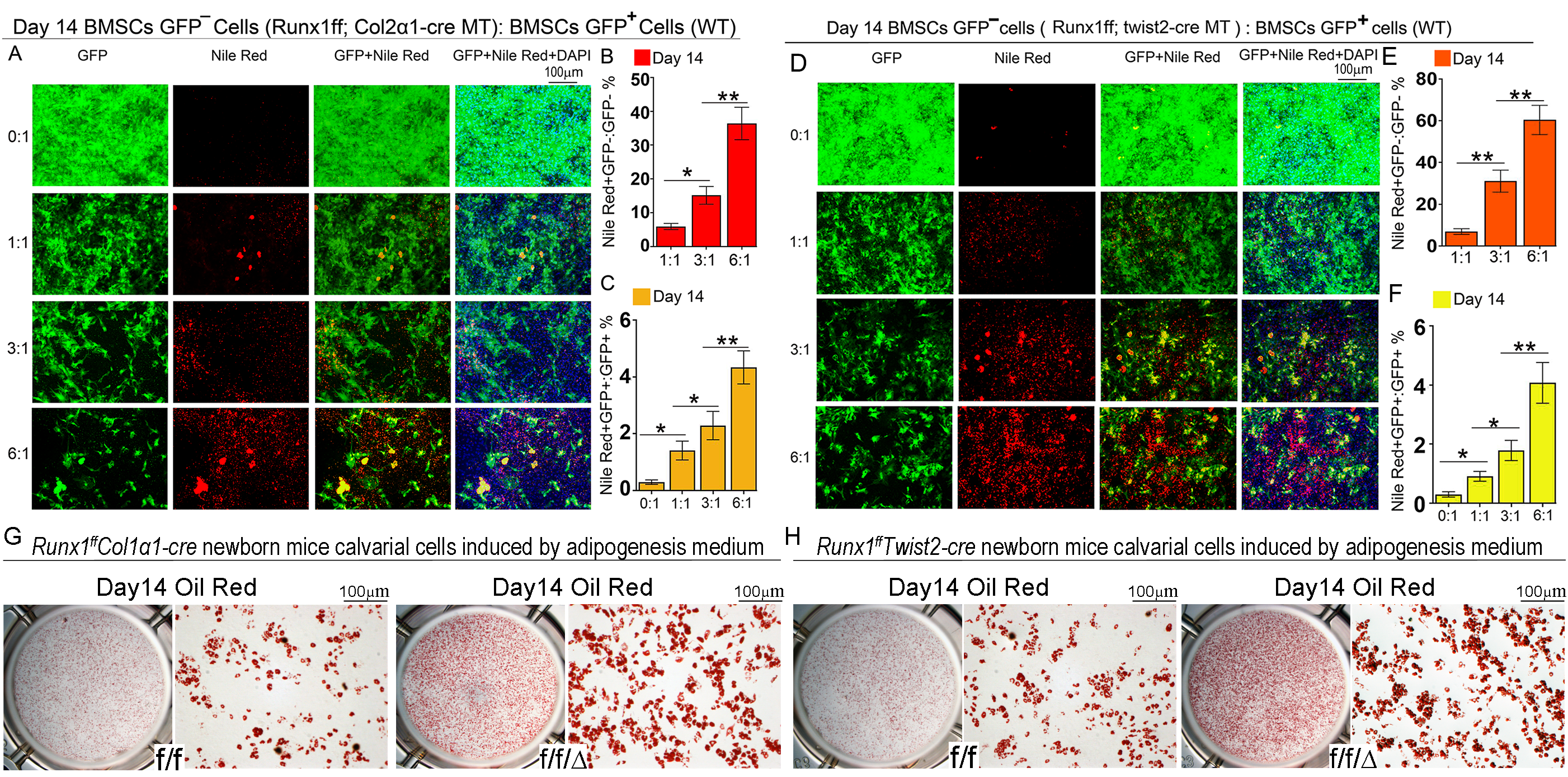

Supplement: S5 Fig — (A-C) Runx1f/fCol2α1-Cre; GFP- and GFP+ bone marrow MSCs were mixed together in different ratios and cultured in osteogenic medium. (A) Adipocytes were labeled by Nile Red and couterstained by DAPI on Day 14. Quantification of (B) Nile Red+GFP-/GFP- and (C) Nile Red+GFP+/GFP+ ratios in A. (D-F) Runx1f/fTwist2-Cre;GFP- and GFP+ bone marrow MSCs were mixed together in different ratios and cultured in osteogenic medium. (D) Adipocytes were labeled by Nile Red and couterstained by DAPI on Day 14. Quantification of (E) Nile Red+GFP-/GFP- and (F) Nile Red+GFP+/GFP+ ratios in D. (G) Oil-Red staining of Runx1f/fCol2α1-Cre and (H) Runx1f/fTwist2-Cre calvarial cells adipogenesis induction medium for 14 days. The data were presented as mean ± SD, n = 8. *p < 0.05, **p < 0.01. (TIF) [file pgen.1009233.s005.tif]

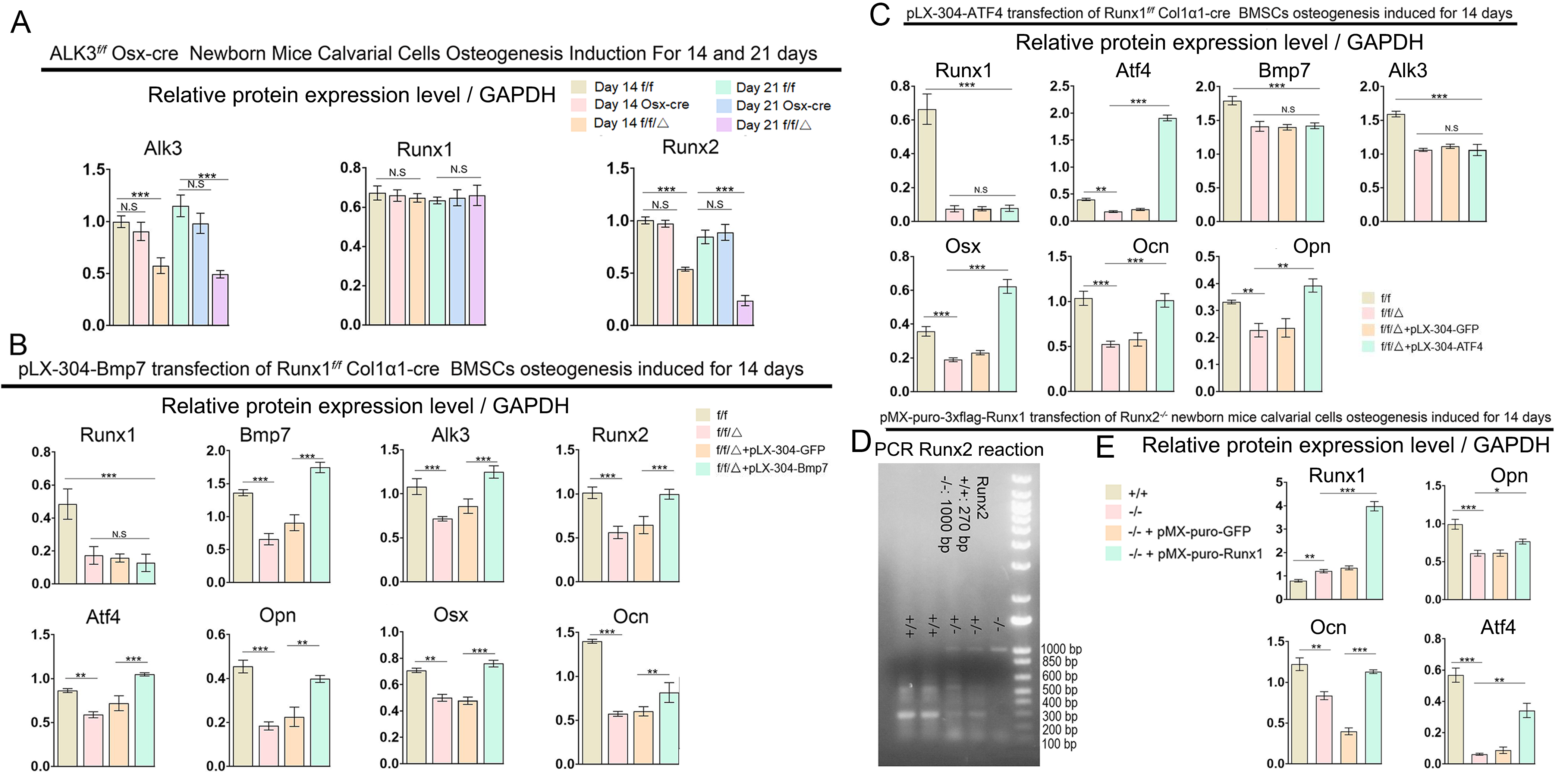

Supplement: S6 Fig — (A) Quantification of western blot data in Fig 5B. (B) Quantification of western blot data in Fig 5D. (C) Quantification of western blot data in Fig 5F. (D) PCR was used to determine Runx2 alleles (f/f, f/+, +/+, or deletion). (E) Quantification of western blot data in Fig 5H. All data are presented as mean ± SD, n = 3, N.S denotes not significant. *p < 0.05, **p < 0.01, ***p < 0.001. (TIF) [file pgen.1009233.s006.tif]

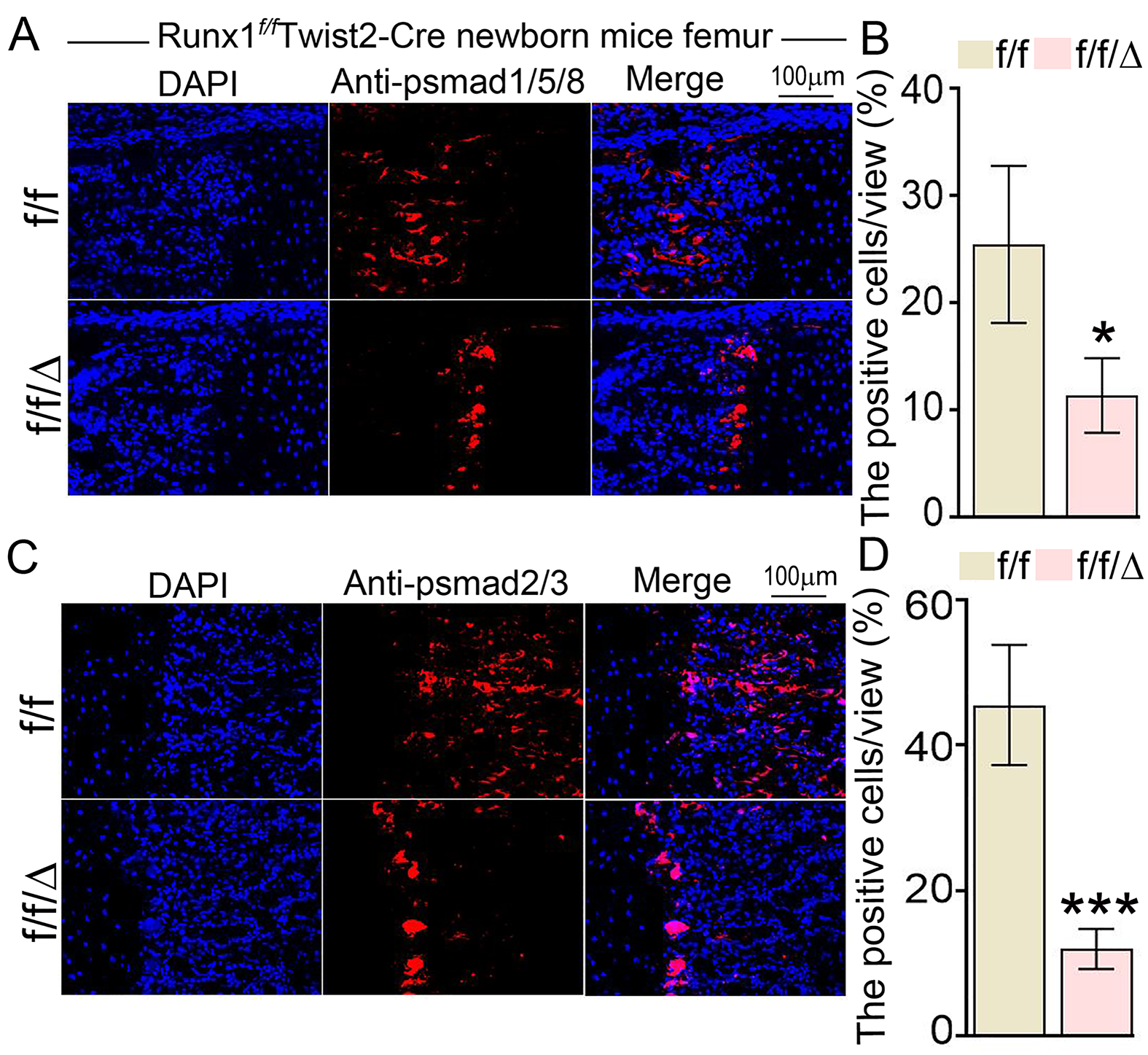

Supplement: S7 Fig — (A) Immunofluorescence staining of anti-phosphorylated Smad1/5/8 in Runx1f/fTwist2-Cre newborn mice femur compared to its control. (B) Quantification data of (A). (C) Staining of anti-phosphorylated Smad2/3 in Runx1f/fTwist2-Cre newborn mice femur compared to its control. (D) Quantification data of (C). All data are presented as mean ± SD, n = 3, *p < 0.05, ***p < 0.001. (TIF) [file pgen.1009233.s007.tif]
