# Supplementary material for: Runx1 is a central regulator of osteogenesis for bone homeostasis by orchestrating BMP and WNT signaling pathways
Source: PLoS Genet. 2021 Jan 21;17(1):e1009233. doi: 10.1371/journal.pgen.1009233 (PMC7819607; doi:10.1371/journal.pgen.1009233)
Supplement: S1 Text — (DOC) [file pgen.1009233.s011.doc]

**Supplemental Materials and Methods**

**Generation of *Runx1^ff^CKO*, *Alk3^ff^Osx-cre,* and *Runx2^-/-^* Mice**

All animal experimentation was carried out according to the legal requirements of the Association for Assessment and Accreditation of the Laboratory Animal Care International and the University of Alabama at Birmingham Institutional Animal Care and Use Committee. Jackson Laboratory, strain name B6.129P2- Runx1tm1Tani/J, JAX no. 008772 were crossed with skeletal tissue cell (MSCs and osteoblasts) using the *Twist2-Cre,* *Col1α1-Cre* (2.3 kb) and *Col2α1-Cre* mouse lines, respectively. *Twist2-cre* mouse line was from Jackson Laboratory, strain name, B6.129X1-Twist2tm1.1(cre)Dor/J, JAX no. 008712). *Col1α1-Cre* (2.3 kb) mouse line was kindly provided by Dr. Crombrugghe from the University of Texas, Houston, TX 77030. *Col2α1-Cre* mouse line was from Jackson Laboratory, strain name B6;SJL-Tg(Col2a1-cre)1Bhr/J, JAX no.003554. *Runx1^f/f^* mice and *Alk3^f/f^* [1] mice with tissue specific promoter-driven Cre were crossed to generate heterozygous mice, which were intercrossed to obtain homozygous CKO mice. *Alk3* strain mouse was kindly provided by Dr. Kai Jiao from the University of Alabama at Birmingham (UAB). *Runx2* heterozygous mice (*Runx2^+/-^*) intercrossed to obtain homozygous mice (*Runx2^-/-^*). All mice were maintained under a 12-h light–dark cycle with ad libitum access to regular food and water at the UAB Animal Facility. Both male and female mice of each strain were randomly selected into groups of five animals each. The investigators were not blinded during allocation, animal handling, and endpoint measurements. The study was approved by the UAB Animal Care and Use Committee, conformed to National Institutes of Health guidelines, and followed all recommendations of Animal Research: Reporting in Vivo Experiments guidelines.

**Histology and Tissue Preparation**

Histology and tissue preparation were performed as described previously [2]. Murine femurs and tibiae were harvested, skinned, and eviscerated before fixing in 4% paraformaldehyde (PFA) in 1×PBS overnight. Samples were then dehydrated in ethanol and decalcified in 10% EDTA for 3 wk. For paraffin sections, samples were dehydrated in ethanol, cleared in xylene, embedded in paraffin, sectioned at 6μm with a Leica microtome, and then mounted on Superfrost Plus slides (Fisher). For frozen sections, samples were infiltrated in 30% sucrose, embedded in optimum cutting temperature (OCT) compound, sectioned at 8 μm with a freezing microtome, and then mounted on Superfrost Plus slides (Fisher).

**H&E and IHC Staining**

H&E staining was performed as described previously [2]. Mice were skinned and eviscerated, and then fixed in 4% PFA overnight. Specimens were dehydrated in ethanol and embedded in paraffin. Sections were cut at a thickness of 6 μm with a microtome and then mounted on Superfrost Plus slides (Fisher). Sections were deparaffinized and hydrated through a xylene and graded ethanol series, rinsed in hematoxylin, in 1% acid alcohol and ammonia-H2O, and then in eosin. Slides were dehydrated in graded ethanol and xylene. The 6-μm paraffin sections were deparaffinized, and antigens retrieval was achieved by heat treatment with a commercial reagent (Abcam AB970). Immunohistochemistry staining was performed using a commercial kit (Mouse on Mouse Basic kit CAT#BMK-2202; Vector Laboratories) and mouse anti-Runx1 (sc-101146; Santa Cruz), goat anti-Runx2 (sc-12988; Santa Cruz), rabbit anti-Atf4 (sc-200; Santa Cruz), rabbit anti-Opn (ab8448; Abcam), mouse anti-Ocn (sc-365797; Santa Cruz) and rabbit anti-Opn (ab8448; Abcam). Mice femurs and tibias were analyzed by immunofluorescence using the following primary antibodies: rabbit-anti-pSmad1/5/9 (#13820S; Cell signaling), rabbit-anti-pSmad2/3 (#8828S; Cell signaling); and these secondary antibodies: TR-goat-anti-rabbit IgG (H+L). The procedure followed the manufacturer’s instructions. Slides were counterstained by hematoxylin.

**Cell Culture and Osteoblast Function.**

Primary calvarial osteoblasts were isolated from newborn mice and BMSCs and seeded in culture at 3×10^3^ cells per square centimeter as described [3, 4]. Subsequently, cells were induced using osteogenic medium and BGJb medium (12591; Gibco) supplemented with 10% (vol/vol) FBS, 50 μg/mL L-ascorbic acid (A4544; Sigma-Aldrich), and 5 mM glycerol phosphate (G9891; Sigma-Aldrich). Bone marrow MSCs were isolated as described [5]. Cells were passaged and osteoblastogenesis were induced using osteogenic medium (α-MEM medium supplemented with 50 μg/mL L-ascorbic acid, 5 mM β-glycerol phosphate, and Dexamethasone). Osteoblastogenesis was analyzed by ALP staining according to manufacturer’s manual (A2356; Sigma-Aldrich). Osteoblast mineralization was examined by Von Kossa staining. Oil-red staining was conducted in cultured dishes as previously descried [4].

**Western Blot Analysis.**

Protein samples were prepared from calvaria-derived osteoblasts and BMSCs in protein lysis buffer as described [2, 6].. Proteins were resolved on SDS/PAGE and electrotransferred on nitrocellulose membranes. Osteoblast- regulators and marker genes including Runx1, Cbfβ, Runx2, Osterix, Atf4, Ocn, Opn, Bmp7, Alk3 and GAPDH were detected using primary antibodies as follows: rabbit anti-Runx1 (8529s; Cell Signaling), rabbit anti-cbfb (61284s; Cell Signaling) rabbit anti-Runx2 (12556s; Cell Signaling), rabbit anti-Sp7/Osterix (ab22552; Abcam), rabbit anti-Atf4 (sc-200; Santa Cruz), mouse anti-Ocn (sc-365797; Santa Cruz), rabbit anti-Opn (ab8448; Abcam), mouse anti-Bmp7 (ST1631; Millipore), rabbit anti-Alk3 (ABD51; Millipore), mouse anti-GAPDH (sc-25778; Santa Cruz) and mouse–anti–β-tubulin (E7; DSHB). Horseradish peroxidase-linked anti-rabbit IgG (7074) and Horseradish peroxidase-linked antimouse IgG (7076) were from Cell Signaling.

**qRT-PCR Analysis.**

Total RNA was isolated from cultured cells at day 7 and day 14 (as indicated) with TRIzol reagent (15596018; Life Technologies). Mouse cDNA was reverse-transcribed from 0.5 g total RNA with SuperScript VILO Master Mix (11755050; LifeTechnologies). The qRT-PCR was performed using the one step RT-PCR System as previously described [2, 6]. Primer sequences are presented in the Table S1.

**Nile Red Staining.**

Differentiated cells are fixed by paraformaldehyde, rinsed by PBS, stained by 25 ng/mL Nile Red, counterstained by DAPI, and visualized by fluorescent microscope.

**Chromatin Immunoprecipitation**

Chromatin Immunoprecipitation (ChIP) was performed as described using primary osteoblast lysates [3]. After immunoprecipitation using rabbit polyclonal anti Runx1 antibody (ab23980; Abcam) and DNA extraction, quantitative PCR was performed using the primers in the promoter region of mouse Bmp7, Alk3 and Atf4 genes (primer sequences are presented in the Table S2).

**Promoter Luciferase Assay.**

The promoter region (−) and (+) of the mouse Bmp7, Alk3 and Atf4 gene was amplified by PCR using Bmp7, Alk3 and Atf4 Bac clone (cat#CH29-27K23; CHORI). Primer sequences are available in Table S3. Then the promoter regions were inserted into the pGL3-basic vector to construct the pGL3-Bmp7, Alk3 and Atf4 promoter vectors and respectively. The insertions of the constructs were confirmed by sequencing. C3H10T1/2 cells were cultured in 24-well plates, and were transiently transfected with a DNA mixture containing the pGL3- Bmp7, Alk3 and Atf4 construct respectively (0.3μg) and β-GAL-expressing plasmids (0.03 μg using Lipofectamine and Plus reagents. Luciferase was detected using Glo Luciferase Assay System (Promega) 48 h post transfection as described [3]. The β-GAL activity of the cell lysates was analyzed using β-Galactosidase Enzyme Assay System (E2000; Promega). The level of luciferase activity was normalized to the level of β-GAL activity.

**Virus production and infection.**

For retrovirus production, pMXs vectors was transfected into 293GPG cells (gift from Dr Xu Feng) and for lentivirus production, pLX-304 vectors was transfected into 293T cells, using a calcium phosphate co-precipitation method and retrovirus supernatant were harvested between 48–96 h [7]. And lentivirus or retrovirus titers were determined by transfecting HEK293T cells with serial dilutions of virus supernatant. AAV was produced and tittered according to the manufacturer's instructions (Life Technology, Inc). BMSCs were transduced with virus supernatant in the presence of 8 μg ml^−1^ polybrene (Sigma) for 24 h before induced with osteogenesis induction medium.

***RNA-Sequencing Analysis***

Total mRNA was isolated using TRIzol reagent (Invitrogen Corp., Carlsbad, CA) from the osteoblasts that were  cultured  for  14 days in osteogenic differentiation medium following the manufacturer's protocol and was submitted to Admera Health (South Plainsfield, NJ) who assessed sample quality with the Agilent Bioanalyzer and prepared the library using the NEBnext Ultra RNA - Poly-A kit.  Libraries were analyzed using Illumina next generation sequencing and relative quantification was provided by Admera Health.

Read counts were subjected to paired differential expression analysis using the R package DESeq2. Volcano plot of differentially expressed genes was generated using log_2_ (fold change) and –log_10_ (p value) values. Genes were considered significant for upregulation/downregulation if p<0.05. GO analysis was carried out using DAVID online tool ([https://david.ncifcrf.gov/](https://urldefense.proofpoint.com/v2/url?u=https-3A__david.ncifcrf.gov_&d=DwMGaQ&c=o3PTkfaYAd6-No7SurnLt5qpge1aKYwPQyBFS7c8AA0&r=cxp_YfZ8wNq_SJs95qGA9kMRKZv4hgSwKjKrAkbgJv4&m=SfR3jxzoWuyAYsdbNQNo_oD3RlOqN9JXKIhqAiNPW3w&s=p-lUhbjd3bR6ke8JCJkqR0BybDn3Jm_M5-UheKMdTdc&e=)). Top GO downregulated categories were selected according to the P-values and enrichment score, and illustrated as number of genes downregulated in respective category.

Signaling pathway data were analyzed through the use of IPA (QIAGEN Inc.,[https://www.qiagenbioinformatics.com/products/ingenuitypathway-analysis](https://urldefense.proofpoint.com/v2/url?u=https-3A__www.qiagenbioinformatics.com_products_ingenuitypathway-2Danalysis&d=DwMGaQ&c=o3PTkfaYAd6-No7SurnLt5qpge1aKYwPQyBFS7c8AA0&r=cxp_YfZ8wNq_SJs95qGA9kMRKZv4hgSwKjKrAkbgJv4&m=SfR3jxzoWuyAYsdbNQNo_oD3RlOqN9JXKIhqAiNPW3w&s=MxyeT3cpO8ySe0ykQjrGH11oqgkCObY2k6ezBxo7C1w&e=)). We used online heatmap generator tool ([http://www.heatmapper.ca/expression/](https://urldefense.proofpoint.com/v2/url?u=http-3A__www.heatmapper.ca_expression_&d=DwMGaQ&c=o3PTkfaYAd6-No7SurnLt5qpge1aKYwPQyBFS7c8AA0&r=cxp_YfZ8wNq_SJs95qGA9kMRKZv4hgSwKjKrAkbgJv4&m=SfR3jxzoWuyAYsdbNQNo_oD3RlOqN9JXKIhqAiNPW3w&s=WHvdrHXJINfURnoIHSUcdlCL-wGOCfLoPWwVT8f18mE&e=)) to graph the differential expression genes analyzed by IPA.

**OVX-induced bone destruction and AAV-Runx1 and AAV-Cbfβ treatment.**

OVX or sham was performed on two-month-old female mice. One week later and two weeks later those mice were administered with a local calvarial injection of 30ul AAV (titer 10^9-10^/ml) expressing YFP, Runx1, or Cbfβ. Mice were harvested 5 weeks after OVX operation and ﬁxed in 4% PFA. Calvaria bone were analysed by X-ray, u-CT and whole-mount TRAP staining. Calvarial were also decalciﬁed for 3 days, immersed in 30% sucrose overnight and then submitted to frozen section and ALP staining.

**Statistical Analysis.**

The number of animals used in this study was determined in accordance with power analysis and our previous studies [2, 6]. In brief, our study used five mice per group per experiment. Data are presented as mean ± SD (n ≥ 3). Statistical significance was assessed using Student’s t test. Values were considered statistically significant at *P* < 0.05. Results are representative of at least three individual experiments. Figures are representative of the data.

**Supplemental References**

1. Mishina Y, Hanks MC, Miura S, Tallquist MD, Behringer RR. Generation of Bmpr/Alk3 conditional knockout mice. Genesis. 2002;32(2):69-72. Epub 2002/02/22. doi: 10.1002/gene.10038. PubMed PMID: 11857780.

2. Chen W, Zhu G, Hao L, Wu M, Ci H, Li Y-P. C/EBPα regulates osteoclast lineage commitment. Proceedings of the National Academy of Sciences. 2013. doi: 10.1073/pnas.1211383110.

3. Chen W, Ma J, Zhu G, Jules J, Wu M, McConnell M, et al. Cbfβ deletion in mice recapitulates cleidocranial dysplasia and reveals multiple functions of Cbfβ required for skeletal development. Proc Natl Acad Sci U S A. 2014;111(23):8482-7. doi: 10.1073/pnas.1310617111. PubMed PMID: PMC4060659.

4. Wu M, Wang Y, Shao JZ, Wang J, Chen W, Li YP. Cbfbeta governs osteoblast-adipocyte lineage commitment through enhancing beta-catenin signaling and suppressing adipogenesis gene expression. Proc Natl Acad Sci U S A. 2017;114(38):10119-24. Epub 2017/09/03. doi: 10.1073/pnas.1619294114. PubMed PMID: 28864530; PubMed Central PMCID: PMCPMC5617241.

5. Soleimani M, Nadri S. A protocol for isolation and culture of mesenchymal stem cells from mouse bone marrow. Nat Protocols. 2009;4(1):102-6.

6. Wu M, Chen W, Lu Y, Zhu G, Hao L, Li YP. Galpha13 negatively controls osteoclastogenesis through inhibition of the Akt-GSK3beta-NFATc1 signalling pathway. Nature communications. 2017;8:13700. Epub 2017/01/20. doi: 10.1038/ncomms13700. PubMed PMID: 28102206; PubMed Central PMCID: PMCPMC5253683.

7. Ory DS, Neugeboren BA, Mulligan RC. A stable human-derived packaging cell line for production of high titer retrovirus/vesicular stomatitis virus G pseudotypes. Proc Natl Acad Sci U S A. 1996;93(21):11400-6. Epub 1996/10/15. doi: 10.1073/pnas.93.21.11400. PubMed PMID: 8876147; PubMed Central PMCID: PMCPMC38069.
